# Three-dimensional gait analysis of orthopaedic common foot and ankle joint diseases

**DOI:** 10.3389/fbioe.2024.1303035

**Published:** 2024-02-22

**Authors:** Yifan Wang, Yansong Qi, Bingxian Ma, Haihe Wu, Yongxiang Wang, Baogang Wei, Xinghua Wei, Yongsheng Xu

**Affiliations:** Orthopedic Center (Sports Medicine Center), Inner Mongolia People’s Hospital, Hohhot, China

**Keywords:** gait analysis, biomechanical, kinematics, kinetics, multisegment foot model

## Abstract

Walking is an indispensable mode of transportation for human survival. Gait is a characteristic of walking. In the clinic, patients with different diseases exhibit different gait characteristics. Gait analysis describes the specific situation of human gait abnormalities by observing and studying the kinematics and dynamics of limbs and joints during human walking and depicting the corresponding geometric curves and values. In foot and ankle diseases, gait analysis can evaluate the degree and nature of gait abnormalities in patients and provide an important basis for the diagnosis of patients’ diseases, the correction of abnormal gait and related treatment methods. This article reviews the relevant literature, expounds on the clinical consensus on gait, and summarizes the gait characteristics of patients with common ankle and foot diseases. Starting from the gait characteristics of individuals with different diseases, we hope to provide support and reference for the diagnosis, treatment and rehabilitation of clinically related diseases.

## 1 Introduction

Walking is the most important mode of transportation of the human body. It is integrated into daily life and movement and is necessary for human survival. Its process is extremely complex and requires the coordinated deployment of various parts of the body: the movement of the centre of gravity of the body, the flexion and extension of the joints, and the rotation of the pelvis. It is a random movement. Gait is a behavioural feature of human walking that is affected by many factors, such as living habits, nature of work, sex and disease. It is generally believed that gait deviation is often caused by specific neurological, muscle or skeletal pathological features (Baker et al., 2016). Gait analysis is a biomechanical research method that integrates human anatomy, physiology and mechanics to analyse the function and state of human walking. Through kinematic observation and dynamic research, limb and joint actions during human walking are analysed, and the relevant geometric curves and corresponding parameters are depicted. The purpose of disease diagnosis, efficacy evaluation and rehabilitation guidance has been achieved. Gait analysis originated in the 17th century. Giovanni Borelli first proposed the application of mechanics and geometry principles to explore the movement of the musculoskeletal system during exercise. In 1988, the successful development of the VICON system and AMASS hardware gradually brought computer 3D gait analysis technology to people’s attention. The three-dimensional ground reaction force (GRF) was measured by a force plate, and kinematic information and muscle function information were added for further analysis (Sutherland, 2001; Sutherland, 2002; Sutherland, 2005). The purpose of describing the gait pattern of a specific population, evaluating and classifying the functional severity of a specific population, and evaluating the effect of specific treatment interventions has been achieved (Debi et al., 2017). As the technology has continued to improve, gait analysis has been widely used to identify abnormal gait characteristics due to a variety of diseases and to monitor and evaluate these characteristics over time to achieve effective tailored treatment, provide information for the evaluation of predictive results, and better ensure the overall practice of precision medicine. We searched the Web of Science and PubMed databases for literature related to human gait analysis. The search terms included “gait”, “walking”, “biomechanics”, “kinematics”, “foot”, “ankle”, “plantar pressure”, “segment”, and “modelling” combined with the terms analysis, evaluation, and diagnostic techniques. We also used the combined terms of gait analysis, motion analysis, and biomechanic analysis. The retrieved articles were screened for their relevance to gait analysis of common orthopaedic foot and ankle disorders, and references unrelated to gait analysis were excluded. The search for references was limited to the English language, and other older references known to us were also included. In this review, we summarize the application of different gait analysis techniques and foot models in the diagnosis, therapeutic assessment of orthopaedic common foot and ankle disorders.

## 2 Basic concepts of gait analysis

**FIGURE 1:**
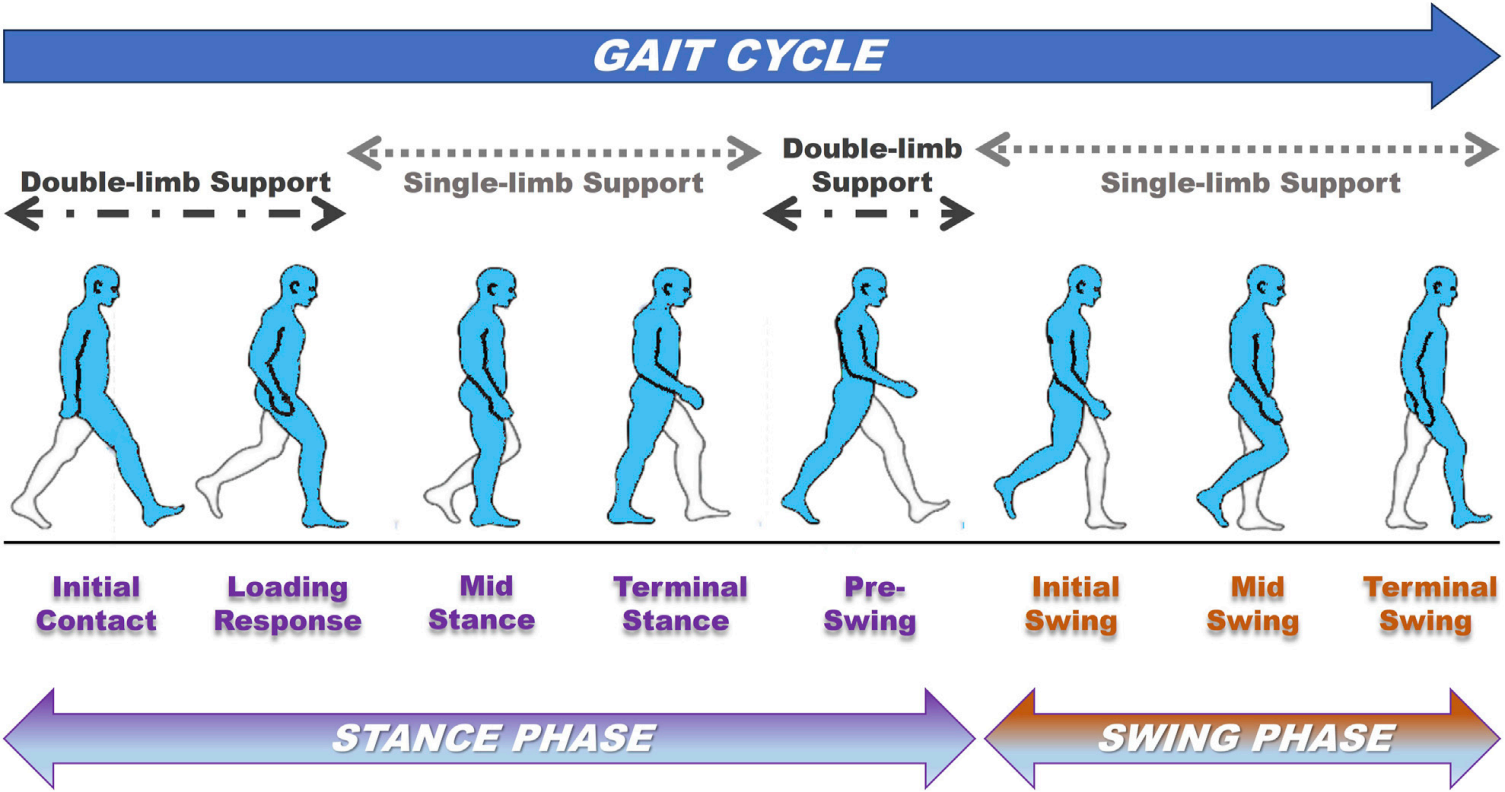
The key phases, stages, and events of the gait cycle.

The gait cycle consists of two main phases: the stance phase and swing phase. During the whole gait cycle, the time that each limb remains in contact with the ground accounts for approximately 60% of the total time, which is called the stance phase. The swing phase accounts for 40% of the movement, which refers to the period when the limb is not touching the ground and is swinging forward (Hecht et al., 2022). The above two stages can be further divided into initial contact, loading response, mid-stance, terminal stance, preswing, initial swing, mid-swing, and terminal swing (Figure 1). The main characteristics of a healthy gait are a stable stance phase, appropriate stride length and step size, prepositioning when swinging, and relatively less energy consumption (Alam et al., 2017). As a dynamic result of the coordination between the nervous system and skeletal muscles, any change in pathological conditions will lead to abnormal gait. The biomechanical study of gait analysis includes a series of biomechanical variables: surface electromyography (sEMG), spatiotemporal parameter, kinematic and dynamic data (Kuo and Donelan, 2010). To help explain these numerous gait parameters, gait indices, such as the gait contour score (GPS) and gait deviation index (GDI), were developed to more clearly reflect gait quality by eliminating subjective differences in the selection of these parameters (Schwartz and Rozumalski, 2008; Baker et al., 2009).

## 3 Foot model and indices

In traditional clinical gait analysis, the foot is modelled as a single rigid part interacting with the tibia, that is, a single-segment model (Brodsky et al., 2011). The advantage of this modelling approach is that it can provide data on the dynamics of the ankle joint (i.e., force, torque and power). However, viewing the foot as a single component is an oversimplification of its complex structure (Theologis and Stebbins, 2010), and this ignores energy generated/absorbed within the segment, distorts our understanding of joint and muscle-tendon dynamics, and thus misrepresents the function of the foot (Mannoni et al., 2003). With the advancement of gait analysis technology, multisegment foot models (MFMs) can explain the above problems by analysing the relative motion between the internal segments of the foot. A variety of MFMs have been developed (Kadaba et al., 1990; Kidder et al., 1996; MacWilliams et al., 2003; Simon et al., 2006; Leardini et al., 2007; Henley et al., 2008; Wright et al., 2011; Oosterwaal et al., 2011; De Mits et al., 2012; Saraswat et al., 2012; Schallig et al., 2022) (Table 1), and there has been much discussion on the application of these models.

**TABLE 1:** Different multi-segment foot models.

References	Model	Number of segments	Segments	Advantages
Kadaba et al. (1990)	Modified Helen Hayes foot model	**1**	Shank (Tib-Fib)	Can provide information on the ankle kinematics and kinetics
			Foot	
Kidder et al. (1996)	Milwaukee Foot Model (**MiFM**)	**4**	Shank (Tib-Fib)	Marker assumptions require radiographs for calibration
			Hindfoot\Forefoot\Hallux	Proven reliability among different centers
Henley et al. (2008)	Dupont Foot Model (**DFM**)	**4**	Shank (Tib-Fib)	Proven reliability among different centers
			Hindfoot\Forefoot\Hallux	Availability of software for data analysis
Wright et al. (2011)	Modified Oxford Foot Model (**OFM**)	**4**	Shank (Tib-Fib)	Proven reliability among different centers
			Hindfoot\Forefoot\Hallux	Availability of software for data analysis
Leardini et al. (2007)	Rizzoli Foot Model (**RFM**)	**4**	Shank (Tib-Fib)	Includes midfoot marker points and focuses on hindfoot position on the coronal plane
			Hindfoot\Midfoot\ Hallux	
Saraswat et al. (2012)	modified Shriners Hospital for Children Greenville foot model (**mSHCG**)	**4**	Shank (Tib-Fib)	reduced required anatomical marker alignment by minimizing the number of anatomical markers and critical alignment directions appropriate for pediatric subjects
			Hindfoot\Forefoot\Hallux	
Schallig et al. (2022)	Amsterdam Foot Model (**AFM**)	**5/6**	Shank (Tib-Fib)	As a clinically informed multisegmental foot model that minimizes kinematic measurement error, is not specific to a particular patient population or age, and can be used in a wide range of clinical applications and patient populations
			Hindfoot\Midfoot	
			Forefoot (optionally divided into a Medial and Lateral forefoot)\Hallux	
De Mits et al. (2012)	Ghent foot model (**GFM**)	**6**	Shank (Tib-Fib)	Allows for increasing resolution in foot biomechanics of the forefoot
			Hindfoot\Midfoot\Medial forefoot \Lateral forefoot \Hallux	Include hindfoot, midfoot, first ray, and hallux can be used to evaluate the windlass mechanism
MacWilliams et al. (2003)	MacWilliams Model	**9**	Shank (Tib-Fib)	Further refinement of the foot’s internal segments allows for the integration of more information on foot kinematics and dynamics-
			Talus/navicular/cuneiform	
			Cuboid \Calcaneus \Medial forefoot \Lateral forefoot \Medial toes \Lateral toes\Hallux \Talus	
Simon et al. (2006)	Heidelberg Foot Measurement Method (**HFMM**)	**-**	-	The mid and forefoot, the method does not incorporate a standard rigid body model, but applies a descriptive method to assess foot motion parameters that are relevant to the clinician
Oosterwaal et al. (2011)	Glasgow-Maastricht foot model	**26**	A forward dynamic model	The model will contain all of the ligaments and muscles of the foot and ankle. The model will provide insight in function of the foot and leg muscles during gait
			An inverse dynamic model	

The bolded font is an abbreviation of the foot model.

Novak (Novak et al., 2014) described the strengths and limitations of clinically used MFMs; MiFM (Long et al., 2010), OFM (Wright et al., 2011), RFM (Arnold et al., 2013), mSHCG (Saraswat et al., 2013), and HFMM (Kalkum et al., 2016) have been proven to have good reproducibility and are commonly used models when conducting clinical studies. OFM and RFM have been shown to be useful for detecting differences in motion in the coronal plane of athletes with foot deformities, as well as for assessing the risk factors for traumatic injuries (Powell et al., 2013), and the mSHCG has been used to address functional differentiation of the developmental foot from the pathologic foot in children (Saraswat et al., 2012). These models differ in many features, such as type of marker cluster, selection of bony markers, construction of anatomical frameworks, definition of joint rotations, and kinematic differences; however, the main difference lies in the definition and selection of the number of foot segments. Most of the models construct the tibial, hindfoot and forefoot frameworks; however, some models choose to construct the midfoot and lesser toe joints, and even scholars build a 26-segmented foot model through finite element simulations to directly study the internal loads and motions of the complex structure of bones and soft tissues (Oosterwaal et al., 2011). Yoo (Yoo et al., 2022) compared the experimental results obtained with five MFMs in terms of reproducibility: the coronal plane of the OFM, DFM, and mRFM models of HFs for labelling-based analyses was less reproducible than was the cross-sectional plane when compared to the MiFM and mSHCG models, which used the angle of offset; in terms of kinematics, all three planes of the HF segments of the OFM and DFM showed a high degree of similarity, and there was a certain degree of similarity between the DFM, the mRFM, and the OFM in the rotational motion of the HF; however, the MiFM and mSHCG showed inconsistent kinematics and a lower ROM. The authors attributed these differences to the different local coordinate systems constructed by the MFMs and differences in the coordination of the segments with each other and the sensitivity of the models to motion; comparisons between studies of different MFMs are not purely difference analyses but must account for numerous influencing factors.

Different from the plane motion of the knee joint, the foot-ankle complex carries out a kind of “spatial motion”. Compared with the motion axis of the knee joint perpendicular to the three basic planes, the motion axis of the foot exists in three-dimensional space because of the influence of the bony structure. There is a certain angle from each plane. Therefore, the motion of the foot is defined by describing the projection of the motion axis on three planes. For the first time, Morton used this “pronation” to describe the movement of the human foot, which is a motion trend that combines three planes; “pronation/supination” is used to describe the special three-plane motion of the foot (Suciu et al., 2016)^.^ In addition to movement trends, there are characteristic gait indicators that can be used to characterize foot and ankle movements (Mcdonald and Tavener, 1999). The foot progression angle (FPA) is the angle between the gait direction and the line between the heel bone and the second heel bone (Beyaert et al., 2003). It can be used to describe the rotational state of the foot, and abnormalities in the FPA reduce the efficiency of mechanical gait, change the force arm of the lower limb force line, induce ankle injuries, and even exacerbate osteoarthritis of the knee; therefore, the FPA is regarded as an important target for improvement in gait rehabilitation (Schelhaas et al., 2022). The centre of pressure (COP), referred to as the “gait line”, is the centroid of the total number of active sensors for each data sample collected and depicts the spatial and temporal relationship between the pressure distribution and the entire plantar surface (Cornwall and McPoil, 2000). The COP has been widely used for the assessment of foot posture, dynamic foot function, and balance (Buldt et al., 2018a). The joint peak torque is the torsional effect that occurs when a moment is applied to a joint; this torque is an important indicator of ankle strength and is influenced by numerous factors, such as foot-ankle joint position and muscle strength. Measurement of joint torque can be used to assess the strength of foot-ankle muscles and identify the weakest range, thus helping individuals detect in advance some foot deformities associated with muscle weakness and neuromuscular diseases, such as Charcot-Marie-Tooth disease (CMT) (Bombelli et al., 2014), hammertoe deformity (Kwon et al., 2009), Duchenne muscular dystrophy (DMD) and diabetic neuropathy (Bakker et al., 2002; Bus et al., 2009), among others, and to develop more specific preventive strength training for clinical populations. Previous studies have shown that ankle strength is an important factor in walking and balance (Ng and Hui-Chan, 2012; Guillebastre et al., 2013); therefore, early clarification of joint torque allows screening for people at high risk of falls and appropriate interventions to prevent fall complications.

## 4 Application in foot and ankle diseases

The foot and ankle joints are the links between the supporting ground and the lower limbs. They bear a large force and face complex and changeable terrain, which plays an important role in the dynamic function of the whole limb. In recent years, the progress in motion analysis technology has offered higher resolution and increased precision in data acquisition, and the development and application of multisegment foot model has enabled clinicians and scientists to explore the analysis of relative motion between anatomical segments of the foot during gait. In this review, we summarize the use of gait analysis in orthopaedic common diseases from the perspective of deformity lesions, trauma and degenerative lesions. (Table 2).

**TABLE 2:** Gait analysis techniques for different foot and ankle joint diseases.

Disease	Model	Subjects	Gait analysis result	
**Cavus Foot**	Plantar pressure test	Pes cavus: n = 34, Neutral feet: n = 34	The pes cavus showed a significant reduction in their weight-bearing areaand significantly increased pressures under all zones of the forefoot except the fifth metatarsal	Fernández-Seguín et al. (2014)
	MFM	Cavus Group: n = 11, Rectus Group: n = 11, Planus Group: n = 11	The Cavus Group showed increased dorsiflexion and inversion in the hindfoot and increased plantarflexion, valgus, and adduction in the forefoot. The Planus Group had less dorsiflexion, more eversion, and more external rotation in the hindfoot as well as less plantarflexion and increased varus in the forefoot	Kruger et al. (2019)
	A five segment foot model and marker set	Normal: n = 37, Pes cavus: n = 30, Pes planus: n = 30	1.Changes in frontal and transverse plane angles of the hindfoot of the cavus group; 2.less motion of the midfoot in the sagittal and transverse planes during initial contact and midstance in the cavus group; 3.reduced midfoot frontal plane ROM during pre-swing in the planus group	Buldt et al. (2015)
	Plantar foot pressure and sEMG	Pes cavus: n = 10	The custom-made insoles dispersed pressure concentrated by the higher medial longitudinal arch and improved the efficient use of muscles	Choi et al. (2015)
**Planus Foot**	Plantar pressure test	Normal: n = 35, Pes cavus: n = 26, Pes planus: n = 31	The largest differences were between the planus and cavus foot groups in forefoot pressure and force. The peak pressures at the 4th and 5th MTPJs in the planus foot group were lower, and displayed the largest effect sizes	Buldt et al. (2018b)
	OFM	Asymptomatic neutral: n = 88, Asymptomatic mild flatfoot: n = 47, Asymptomatic flatfoot: n = 29, Symptomatic flatfoot: n = 30	Hindfoot eversion was increased in children with asymptomatic and, to a greater extent, symptomatic flatfoot. The forefoot was significantly more abducted in the symptomatic and in the flat group. The forefoot was more supinated relative to the hindfoot in the flatfoot groups	Kerr et al. (2015)
	Plug-in gait (PIG) and OFM	Asymptomatic neutral: n = 98, Asymptomatic mild flatfoot: n = 47, Asymptomatic flatfoot: n = 29, Symptomatic flatfoot: n = 38	The symptomatic flat feet showed significant differences from asymptomatic groups (most commonly the neutral feet) in terms of hip flexion, knee flexion and varus, hindfoot inversion-eversion, and forefoot abduction-adduction	Kerr et al. (2019)
	Modified PIG and MSK	Flatfoot: n = 15	The second peak patellofemoral contact force and the peak ankle contact force were significantly lower in the WSFO group. The foot orthosis significantly reduced the peak ankle eversion angle and ankle eversion moment; however, the peak knee adduction moment increased	Peng et al. (2020)
**Congenital Talipes Equinovarus**	Plantar pressure test	NCF who underwent Ponseti treatment: n = 22, Healthy children n = 22	In the present study, a higher proportion of the internal FPA was found in the affected clubfeet. The affected feet showed a significant increase in CA% and a higher PP in the M5 and MF zones. Internal foot progression angle and a load transfer from the medial forefoot and hindfoot to the lateral forefoot and midfoot were observed in the affected feet.	Xu et al. (2018)
	Extended Helen-Hayes model and OFM	The group of successfully treated clubfoot (the nonrelapse group), Relapse clubfoot (the relapse group)	Clubfoot patients with a relapse show lower total gait quality (GDI*) and lower clinical status defined by the CAP. Abnormal cFDI* was found in relapse patients, reflected by differences in corresponding variable scores. Moderate relationships were found for the subdomains of the CAP and total gait and foot quality in all clubfoot patients	Grin et al. (2022)
	Extended Helen-Hayes model and OFM	Control group: n = 15, Corrected group: n = 11, Relapse group: n = 11	The relapse group showed significantly increased forefoot adduction in relation with the hindfoot and the tibia. This group showed increased forefoot supination in relation with the tibia during stance, whereas during swing increased forefoot supination in relation with the hindfoot was found in patients with relapse clubfoot	Grin et al. (2021)
	Helen Hayes model	Dynamic supination (recurrent clubfoot): n = 17	The postoperative step length, stride length, postoperative peak internal ankle rotation angle in the frontal plane, postoperative peak internal foot progression angle in the transverse plane and V-angle-S values were significantly smaller than their preoperative values	Li et al. (2022)
	Cleveland clinical model and OFM	Recurrent clubfoot: n = 17, Healthy childr: n = 25en: n = 18	After TATT, forefoot supination in relation to the hindfoot and tibia was reduced during swing and at initial contact, the heel showed less dynamic varus and adduction movement, Maximum ankle dorsiflexion slightly increased. Maximum ankle power was reduced preoperatively and postoperatively compared with controls	Mindler et al. (2020)
	Helen Hayes model	Relapsed clubfeet: n = 17, Clubfeet without relapse: n = 28	There was statistically significant difference in the parameters of foot length, stride length, and single limb support time (%gait cycle) between the 2 groups	Liu et al. (2020)
**Hallux Valgus**	Plantar pressure test	A population-based study	Participants with HV had lower hallucal loading and higher forces at lesser toes as well as higher MAI and lower CPEI values compared to the referent. Participants with HV and other FDs were also noted to have aberrant rearfoot forces and pressures	Galica et al. (2013)
	OFM	Hallux valgus participants: n = 20, Symptom-free volunteers: n = 22	In our HV population we found an increased dorsiflexion motion at the hallux during terminal stance. In both sub-phases of stance, the HV group showed increased eversion of the hindfoot, indicating a less stable foot	Deschamps et al. (2010)
	DFM	Female symptomatic HV patients n = 58, female nonsymptomatic older volunteers n = 50	For temporal parameters, gait speed and stride length were diminished according to the severity of HV deformity. Sagittal range of motion of hallux and hindfoot decreased significantly and loss of push‐off during the preswing phase was observed and forefoot adduction motion during terminal stance was decreasedin SHV group	Kim et al. (2020)
	Pressure insoles and five 3-D inertial sensors connected with two data-loggers	Female patients with moderate to severe hallux valgus who underwent modified Lapidus procedure: n = 15	Three spatiotemporal, two kinematics, and seven plantar pressure parameters significantly improved between 6 months and 12 months postoperatively. Significant improvement in radiological and clinical outcome was reported at 6 and 12 months	Moerenhout et al. (2019)
	Plantar pressure test	Consecutive feet with postoperative transfer metatarsalgia: n = 30, Feet without metatarsalgia: n = 30	For pain group, the maximum plantar force and force time integral of the first metatarsal decrease significantly, the time point when central rays reached their peak force during the push-off is significantly later than that in controls. The regional instant load percentage at this moment presented significantly higher for central rays, while significantly lower for the first metatarsal and the hallux compared to the controls	Geng et al. (2017)
**Acute Ankle Sprains and Chronic Ankle Instability**	Plantar pressure test and sEMG	CAI patients: n = 17, Healthy subjects: n = 17	The CAI group demonstrated a more lateral COP throughout the stance phase and significantly increased peak pressure and pressure–time integral under the lateral forefoot. The CAI group had higher gluteus medius sEMG amplitudes during the final 50% of stance and first 25% of swing	Koldenhoven et al. (2016)
	vGRF data were collected using an instrumented treadmill	CAI patients: n = 11, Healthy individuals: n = 13	The CAI group had higher impact peak forces, active peak forces, an increased loading rate and a shorter time to reach the active peak force compared with the control group	Bigouette et al. (2016)
	Gait analysis, Musculoskeletal model. Finite element model	Acute LAS participants n = 68, Noninjured participants: n = 19	During period 1, the LAS group displayed increased knee flexion with increased net extensor pattern at the knee joint, increased ankle inversion with a greater inversion moment, and reduced ankle plantar flexion. During period 2, the LAS group displayed decreased hip extension with a decrease in the flexor moment at the hip, and decreased ankle plantar flexion with a decrease in the net plantar flexion moment	Doherty et al. (2015)
	Codamotion marker set model (Winter 2009)	Acute LAS: n = 68, Control group: n = 19	Controls demonstrated greater angles of SAK/TT than individuals with CAI and greater angles of FAK/TT than copers during the second half of stance	Kwon et al. (2020)
	Plantar pressure test and sEMG	CAI patients: n = 16	Gait training improved self-reported function and caused a medial shift in the COP from 10% of stance through toe-off. The medial shift in COP was driven by concurrent increases in peroneus longus muscle activity from 21% to 60% and 81%–90% of stance. There was a corresponding reduction in gluteus medius muscle activity during 71%–100% of stance	Feger et al. (2018)
	Plantar pressure test and sEMG	CAI patients were treated with anatomic reconstruction surgery: n = 19	Dynamic pedography showed a large degree of symmetry of plantar pressure distribution after surgery. There were no significant differences in peroneal reaction time in the repaired and intact ankles	Schmidt et al. (2005)
**Ankle Fracture**	Plantar pressure test and the spatiotemporal gait parameters	Patients with bimalleolar ankle fractures undergoing ORIF: n = 22, Healthy subjects: n = 11	The main results found in plantar pressure were a lower mean/peak plantar pressure, as well as a lower contact time at 6 and 12 months with respect to the healthy leg and control group and only the control group, respectively. In the ankle fracture groups there are a moderate negative correlation between plantar pressures (average and peak) with bimalleolar and calf circumference	Fernández-Gorgojo et al. (2023)
	PIG、plantar pressure test and sEMG	Patients with trimalleolar ankle fractures, Healthy subjects: n = 12	Patients presented compromised gait patterns: shorter step length, larger step width, slower walking speed and shorter single support, and asymmetrical gait. During walking, patients showed abnormal dynamic plantar pressure features (mainly in the hindfoot and forefoot regions), and the IEMG of TA and PL were larger than healthy controls	Zhu et al. (2022)
	OFM	Patients operated for ankle fractures: n = 33 (33 feet), Healthy control group: n = 11 (20 feet)	Significantly less ROM between the hindfoot and tibia in the sagittal plane (flexion/extension) during loading and push-off phases was found in patients after ankle fractures. Lowest ROM and poorest PROM results were found for patients with trimalleolar ankle fractures	van Hoeve et al. (2019)
	HFMM	Patients with isolated ankle fractures: n = 14, Healthy participants: n = 20	Significant differences for the Foot Tibia Dorsal Flexion, the tibio-talar dorsal flexion and the ground reaction force for patients after 9 weeks as well as patients after 26 weeks compared to healthy participants, respectively. The ROM in the tibio-talar joint and the medial arch was reduced in affected patients compared to healthy participants	Böpple et al. (2022)
	RFM	Patients who sustained a trimalleolar fracture and underwent surgery: n = 15, Asymptomatic adults: n = 13	Mean peak power generation, total positive work and peak internal ankle moment were significantly lower for the Chopart joint when comparing the patients to the control group. These results were observed for both the affected and unaffected side of the patients, showing symmetrical changes in the patient group	Deschamps et al. (2022)
**Ankle Osteoarthritis**	MiFM	DJD group: n = 36, Healthy group: n = 13	Ankle DJD demonstrates significant changes in foot mechanics characterized by altered segment kinematics and significant reduction in dynamic ROM at the tibia, hindfoot, forefoot, and hallux when compared to controls. The results demonstrate decreased temporal-spatial parameters	Canseco et al. (2018)
	Dynamic pedobarography	Posttraumatic end-stage ankle osteoarthritis patients: n = 120	Maximum force and contact area were decreased in the whole osteoarthritic foot. Peak pressure in the hindfoot and toes area was decreased as well. The results indicated a positive correlation between dorsiflexion and the pedobarographic parameters	Horisberger et al. (2009)
	A 3D MFM with 15 markers (Seo et al., 2014)	Patients undergoing TAR: and AA: n = 17 and 7	Gait speed was faster in the TAR, the range of hindfoot and forefoot sagittal motion was significantly greater in the TAR. The main component of motion increase was hindfoot dorsiflexion. Maximum ankle power in the TAR was significantly higher than in AA. However, the range of hindfoot and forefoot sagittal motion was decreased in both TAR and AA.	Seo et al. (2017)
	mOFM	Patients undergoing TAR: and AA: n = 10 and 10	During level walking, sagittal ankle ROM was significantly higher, forefoot-tibia motion and hindfoot-tibia motion were significantly greater in the TAA group. During stair ascent, sagittal ankle ROM, forefoot-tibia motion, and hindfoot-tibia motion was greater	Sanders et al. (2021)
	MiFM	DJD patients were evaluated before and after TAA: n = 27	Decreased external rotation of the tibia and increased external rotation of the hindfoot were noted throughout the gait cycle. Ankle replacement as supported by increased temporal-spatial parameters, and significant improvement in tibial sagittal range of motion during terminal stance and hindfoot sagittal range of motion during preswing	Fritz et al. (2022)

### 4.1 Cavus foot

Cavus foot deformity is characterized by an increased longitudinal arch of the foot. The aetiology is relatively complex and often combined with varus, horseshoe or other deformities. According to previous studies (Walker and Fan, 1998; Crosbie et al., 2008; Sanchis-Sales et al., 2019), the proportion of cavus foot in the population is approximately 10%–15%, and 60% of patients will experience foot pain. A study of plantar pressure in two-foot positions showed that the plantar contact area was significantly reduced in cavus foot, with the forefoot becoming the main weight-bearing structure and the load distribution shifting towards the medial aspect of the foot (Fernández-Seguín et al., 2014). Of these, the second and third metatarsal heads are under the most pressure, and because the metatarsals are embedded between the cuneiform bone joints, the area has low mobility, making it difficult to distribute the load (De Doncker and Kowalski, 1979), which has been suggested to be the cause of the associated pain. In terms of kinematics, Kruger (Kruger et al., 2019) compared the effects of different foot postures on movement. Compared with those in the normal group, the cavus foot group had obvious hindfoot dorsiflexion and varus, and plantar flexion, valgus and adduction of the forefoot also increased, which was inconsistent with the increase in hindfoot valgus and abduction and increase in forefoot adduction reported by Buldt et al. (2015). The possible reasons for these results are as follows: 1. The segmental models used are different, and the positioning and direction of the coordinate axes are also different. 2. There may be differences in the reference position used (neutral position/comfort position).

Buldt reported that the middle foot coronal mobility of the cavus foot group decreased during the stance period, which indicated that the deformation of the medial longitudinal arch was reduced, the foot became more rigid, and the arch rigidity increased, which may have caused the foot to absorb the impact load. The ability to absorb the impact load can be reduced when a patient with a high arch foot touches the ground, causing damage to the arch. Therefore, in clinical practice, it is necessary to improve the detection, evaluation and early correction of plantar pressure and kinetic parameters in this group of people to reduce pain in the corresponding area and prevent the occurrence of sports injury. Choi et al. (2015) designed an orthopaedic insole containing a metatarsal pad that can reduce foot supination. The results showed that the customized insole can significantly increase the contact area of the middle foot, reduce the outwards heel tilt, improve the pressure distribution of the forefoot, disperse the concentrated pressure at the hindfoot and the forefoot, and improve the effective utilization of the lower-limb muscles to achieve the effect of reducing lower-limb fatigue during long gait.

### 4.2 Planus foot

Flatfoot is a common foot deformity in foot and ankle surgery. It is characterized by the reduction or disappearance of the arch of the foot. In fact, this condition is accompanied by hindfoot valgus, forefoot abduction and supination (Levinger et al., 2010a), which may be related to congenital development, acquired trauma and other factors. Rigid flatfoot (such as tarsal bone fusion and the vertical talus) caused by neuromuscular diseases can cause symptoms such as pain or walking disorders. Flexible flatfoot (FFF) caused by soft tissue relaxation around joints is common in clinical practice, especially in children and adolescents. Although simple flatfoot has little effect on the shape of the foot, it often affects the gait of patients in the late stage. Dynamic gait analysis combined with static imaging analysis is needed for overall evaluation. Compared to cavus foot, planus foot showed opposite plantar pressure characteristics, the group showed greater midfoot contact area along with lower peak pressures in the fourth and fifth Metatarsophalangeal Joint (MTPJ) and lateral heel (Buldt et al., 2018b). An important finding of this study was the distribution of the loads on the structures surrounding the 1st MTPJ, with the planus foot hallux having higher loads and the 1st MTPJ having lower loads (in complete contrast with the cavus foot), this alteration is similar to that of patients with first MTPJ lesions (e.g., hallux rigidus) (Menz et al., 2018), and the authors considered that the first metatarsophalangeal joint function may show a certain tendency to change in relation to the time of the development of arthropathology.

Kerr et al. (2015) compared the differences between planus patients with and without clinical symptoms. The results showed that, compared with those in the normal group, all the planus foot groups exhibited significant hindfoot eversion, and the symptomatic group had significantly more forefoot abduction than the asymptomatic group; moreover, there was a tendency towards forefoot supination relative to the hindfoot. In addition to foot changes, the symptomatic group showed a significant increase in hip flexion when the foot was following the ground, knee flexion in the middle, and knee valgus (Kerr et al., 2019). Due to the increased hindfoot eversion in planus foot patients, the arch of the planus foot increases the flexibility of the foot but is unable to provide the rigid lever required for push-off. This results in an inability to provide support to the soleus and gastrocnemius and a decrease in forward thrust, decreasing stride speed. Moreover, forefoot abduction may lead to excessive tension on medial soft tissue structures, excessive pronation of the hindfoot rotates the tibia, causing passive strain on the foot and changing the direction of patellofemoral joint forces. These potential changes in motion can lead to worsening of conditions and symptoms such as foot pain, plantar fasciitis, fatigue, joint instability and patellofemoral joint pain (Riskowski et al., 2013). Therefore, to improve foot pain, the first task is to restore the arch of the foot, normalize the movement of the foot, and diagnose and treat the patient as soon as possible to avoid decompensation of adjacent joints. Peng et al. (2020) tested a foot orthosis (medial arch support with medial forefoot posting). Compared with ordinary footwear walking, orthosis can significantly reduce the peak ankle contact force, the peak ankle valgus angle and the peak ankle valgus torque; maintain the medial longitudinal arch; prevent further pronation; significantly reduce the second peak of the patellofemoral joint contact force; and relieve patellar joint pain.

### 4.3 Congenital talipes equinovarus

Congenital talipes equinovarus (clubfoot) is the most common congenital foot structure deformity. There is one case in every 1000 newborns worldwide (Ansar et al., 2018). The main manifestations are clubfoot (increased plantar flexion of the calcaneus relative to the tibia), cavus foot (pronation of the forefoot relative to the hindfoot, increased longitudinal arch of the foot) and adduction and varus deformity (increased adduction and/or varus of the calcaneus relative to the tibia) (Ponseti et al., 2006). In clubfoot, more males than females, slightly more unilateral cases than bilateral cases, and seasonal changes in symptoms have been reported. The incidence of clubfoot in autumn and winter is high (Wallander et al., 2006; Zhao et al., 2016), and it is more common in patients with motor neuron injury. There are many differences in the gait characteristics of children with clubfoot compared with those of children with normal development. The main manifestations are a decrease in ankle joint activity, an increase in the internal rotation of the foot, a decrease in ankle joint strength during the swing period, a decrease in plantar flexion torque and a possible foot drop. Compensatory changes, such as increased hip external rotation and excessive knee extension, were also observed. (Mindler et al., 2014; Lööf et al., 2016). The purpose of treating clubfoot is to relieve foot pain, obtain a normal appearance, and restore normal function. At present, the Ponseti method (manipulation, casting, cementing, or an Achilles tenotomy) is considered the gold standard for early treatment (Ganesan et al., 2017; Grin et al., 2023). A transplantar pressure study showed that, compared to those of the healthy side and control group, clubfoot treated with the Ponseti method had higher peak pressure (PP) and pressure‒time integral (PTI) in the lateral forefoot and midfoot; moreover, the contact area reported as a percentage of the total foot area (CA%) was also significantly greater, while the PP and PTI were significantly lower in the medial forefoot and hindfoot, suggesting that the affected foot experiences significantly greater mechanical stress during walking and that the load is transferred from the medial side of the foot to the lateral forefoot and midfoot (Xu et al., 2018). An “in-toe” gait pattern was observed in the affected clubfoot, with an internally rotated FPA that facilitates the transfer of loads to the lateral aspect of the foot.

Although the Ponseti method can achieve good results in the short term, 11%–47% of patients have been reported to experience disease recurrence. Several studies have shown that among patients who have received Ponseti treatment, the disease rate at 3.7–4.1 years after correction is still as high as 68% (Bergerault et al., 2013; Zionts et al., 2018). Patients with recurrent cases showed a similar gait pattern to that before correction (Grin et al., 2021; Grin et al., 2022). Grin et al. (2021) reported that, compared with those in the nonrecurrent group, the forefoot adduction and supination angles (relative to the hindfoot and tibia) in the recurrent group were significantly greater. The authors believe that this may be a kinematic indicator of recurrent talipes equinovarus, which is helpful for early identification of recurrent talipes equinovarus. Gait analysis plays an important role in the early detection of recurrence, and gait analysis technology can also be used to determine and evaluate the effect of additional treatment. Recent studies on the efficacy of tibialis anterior tendon transfer (TATT) and repeated Ponseti treatment have revealed changes in the movement of recurrent patients after additional treatment (Liu et al., 2020; Mindler et al., 2020; Li et al., 2022). TATT is a surgical technique that restores the muscle balance between varus and valgus of the hindfoot by transferring the anterior tibial tendon insertion to the lateral cuneiform bone. Studies have shown that TATT can significantly improve the dynamic supination of the forefoot in recurrent patients and appropriately relieve varus and adduction of the hindfoot, but the postoperative ankle strength decreases to varying degrees (Mindler et al., 2020; Li et al., 2022). This result is related to muscle weakness and atrophy of the affected limb; that is, the operation restores the dynamic imbalance but cannot restore lost muscle mass, and an invasive operation plus long-term postoperative plaster fixation may lead to further muscle atrophy. Liu et al. (2020) compared the kinematic and dynamic parameters of patients with recurrent talipes equinovarus treated with repeated Ponseti with those of patients without recurrence. The results showed that there was no significant difference between the recurrent and nonrecurrent patients treated with Ponseti except for spatial and temporal parameters such as step length and single-limb support time. Therefore, the author suggested that the Ponseti method should be used for recurrent talipes equinovarus.

### 4.4 Hallux valgus

Hallux valgus (HV) deformity refers to the deviation of the great toe from the midline of the foot to the second toe and lateral oblique displacement at the first metatarsophalangeal joint. It can cause the formation of a painful bursa at the medial convex of the great toe and metatarsophalangeal joint, accompanied by symptoms such as forefoot pain and deformity of the remaining toes (Coughlin and Shurnas, 2003). The incidence rate is approximately 23%–35.7%, and the prevalence rate in women (30%) is higher than that in men (13%) (Nix et al., 2010). A large-scale male‒female cohort study of the HV population (Galica et al., 2013) showed that participants with HV alone and HV combined with other foot diseases had lower loads in the toe area and greater loads in the small toe area than participants without HV. In addition, the group with HV had lower hindfoot lateral force, peak pressure and centre of pressure excursion index (CPEI) values and greater modified arch index (MAI) values. These findings indicate that, compared with those in the control group, HV patients in the control group tended to exhibit a more prone gait and may exhibit a flatfoot posture. In terms of kinematics, HV patients tend to exhibit excessive foot dorsiflexion, increased hindfoot valgus, reduced abduction of MTP1, and reduced ankle mobility during walking (Deschamps et al., 2010). Similarly, Kim et al. (2020) et al. studied the effect of HV deformities on the intersegmental motion of the foot. The results showed that the sagittal ROM of the hallux toe and the hindfoot decreased, the hallux toe was overly dorsiflexed at the end of the support phase, and the adduction of the forefoot decreased. These changes were positively correlated with the severity of the deformity.

According to the severity of the deformity, a customized orthosis can be selected to prevent disease progression, and surgical intervention can also be performed. The effect of the surgical plan also differs. Moerenhout et al. (2019). performed gait analysis on the efficacy of modified Lapidus surgery and reported that at 6–12 months after surgery, 3 spatial-temporal parameters, 2 kinematic parameters and 7 plantar pressure parameters were significantly improved. Moreover, the results 12 months after surgery were significantly better than those at 6 months, indicating that the recovery time required after surgery was also relatively longer. In the walking process, whether the remaining area can fully bear a certain load is a problem that cannot be ignored. Ballas et al. (2016). performed a series of studies on the change in plantar pressure after valgus surgery and reported that the gait pattern after scarf surgery was similar to that without surgical treatment, but the forefoot propulsive force could not be fully restored in patients who underwent first metatarsophalangeal arthrodesis. Geng et al. (2017) also reported that the first metatarsal load function was impaired in some patients with metatarsal pain after valgus surgery. Whether the remaining part can bear the original load while walking is a problem that cannot be ignored.

### 4.5 Acute ankle sprains and chronic ankle instability

Acute ankle sprain is the most common lower-limb movement-related injury, accounting for 16%–40% of all sports-related injuries (Halabchi and Hassabi, 2020), and acute ankle sprain is most common in basketball, football and rugby. Repeated ankle sprains and residual symptoms such as persistent instability after injury are called “chronic ankle instability” (CAI) (Waterman et al., 2010). CAI can also be subdivided into functional ankle instability (FAI) due to interruption of the afferent pathway, resulting in functional instability of proprioception defects, and modified arch index (MAI), caused by structural changes in the ankle complex (Delahunt et al., 2006). Due to impaired postural control, the movement strategies of ankle sprains and CAI patients change during walking. Koldenhoven et al. (2016) investigated the plantar pressure characteristics of CAI patients walking on level ground and standing on one foot and reported that the COP on the affected side of CAI patients significantly shifted towards the lateral side of the foot while walking on level ground, and the stability of the affected side in the anteroposterior, posterior, and bilateral internal and external postural control was significantly weaker than that of normal subjects during unipedal support. Bigouette et al. (2016) conducted a cohort study on the changes in the vGRF in CAI patients during running. The study revealed that, compared with healthy controls, the CAI group had a greater peak impact force and active peak force, an increased load rate, and a shortened time to reach the active peak force. This change may increase the vulnerability of CAI patients to stress-related injuries and repeated sprains. These changes may also explain why CAI patients are prone to repeated sprains and confirm that CAI kinematics are more inclined to lead to ankle inversion.

Doherty et al. (2015) analysed the kinetic changes in the limbs before and after heel touchdown (period 1) and after toe-off from the ground (period 2) in patients with lateral ankle sprain (LAS). Compared with those in the healthy control group, in the first stage, knee flexion, ankle varus torque, and varus flexion were greater, while plantar flexion was lower in the LAS group. In the second stage, hip extension and plantar flexion decreased in the LAS group. The injured person protects the fragile ankle joint through local and global adjustments and exhibits many movement patterns similar to those of individuals with CAI. Kwon et al. (2020) studied the coordination of limbs between different groups. The results showed that the CAI and Coper groups exhibited different coordination strategies than did the control group in the second half of standing and in the process of propulsion (i.e., compared with the tibial internal rotation of the healthy population, the experimental group showed tibial external rotation), which further increased the evidence of limb regulation and compensation for sensorimotor deficits.

Treatment of CAI is primarily aimed at improving poor compensatory movement patterns, allowing for a reduction in ankle dorsiflexion and thus enhancing postural control stability. Feger et al. (2018) reported that the COP shifted medially during 10%–100% of the walking cycle after gait training. The contact area of the medial midfoot increased significantly and reached peak pressure 13% earlier than before training, and the PTI of the medial forefoot increased significantly, indicating that gait training could cause the COP to shift inwards earlier and more rapidly and reach a stable ankle position. Approximately 34% of ankle sprain patients develop sprain again within 3 years of injury (van Rijn et al., 2008). If conservative treatment fails and symptoms of chronic ankle instability persist, surgical treatment can be selected by repair (anatomical contraction of the lateral ligament and reconstruction of the insertion point) or reconstruction (reconstruction of the lateral ligament with other surrounding tissues). Schmidt et al. (2005) performed plantar pressure tests on CAI patients 3 years after Karlsson’s anatomical repair. The results showed that there was no significant difference between the peak pressure in 8 plantar regions (toe, 2–5 toes, first metatarsal head, second and third metatarsal head, midfoot, medial hindfoot, lateral hindfoot) and that in the healthy foot during walking. These studies may help clinicians intervene in CAI by reducing the load rate and improving posture training for patients to prevent the recurrence of sprains and slow the progression of osteoarthritis after ankle trauma, which is beneficial for ensuring patient prognosis.

### 4.6 Ankle fracture

Ankle fracture (AF) is the most common fracture in orthopaedic trauma surgery and accounts for approximately 9% of all fracture types, with an annual incidence of 107–184 cases/100,000 people (Court-Brown and Caesar, 2006). The most common fracture types are single and double ankle fractures (Shibuya et al., 2014; Elsoe et al., 2018). Fractures caused by low-energy indirect trauma are generally treated conservatively, but when conservative treatment fails to restore joint consistency and fractures are caused by high-violent trauma, surgical reduction is the preferred treatment method (Goost et al., 2014). The most commonly used surgical treatment is open reduction and internal fixation (ORIF), the main purpose of which is to prevent posttraumatic arthritis and shorten the immobilization time (Tantigate et al., 2019). The severity of the injury, surgical intervention and fixation time all contribute to biomechanical changes (Egol et al., 2006), and recovery of these parameters after ankle treatment is critical. A study of dynamic plantar pressure changes at 6 and 12 months after bilateral ankle fracture surgery showed that AFG patients had lower plantar pressure (peak and mean) and longer contact time than did CG patients and that AFG patients had significantly lower e-cadence and gait speed than did the control group. Similarly, (Fernández-Gorgojo et al., 2023), Zhu et al. (2022) reported similar results in a study of 12 subjects after trimalleolar fracture; i.e., abnormal changes in plantar pressure were mainly manifested in the forefoot and hindfoot regions. Compared with those in the healthy control group, the patients in the symptomatic group had shorter step lengths, larger step widths, slower walking speeds, shorter single supports and obvious asymmetry in gait; these authors believe that these differences might be related to functional impairment of the anterior tibial and peroneal longus muscle ability.

van Hoeve et al. (2019) performed gait analysis on ankle fractures of different severities treated by surgery. The results showed that the patient’s sagittal mobility in weight-bearing and push-off segments was significantly reduced, but there was no obvious abnormality in cross-sectional or coronal movements. The author emphasized that the severity of the fracture is inversely proportional to the extent of joint flexion/extension. Similarly, Böpple and his colleagues (Böpple et al., 2022) reported that the motion of the tibiotalar joint and medial arch in patients with ankle fractures was limited, and the regional GRF was also reduced; however, over time, significant improvement could be observed. The author also confirmed that patients with fractures were more inclined to lift their feet rather than push them off the ground during forward movement. Deschamps et al. (2022) quantified the foot joint mechanics (joint torque) and energy (power) of patients walking after ORIF and noted that, compared with those in the control group, all components of the ankle complex in the ORIF group showed varying degrees of reduction. Among them, the peak value of the medial malleolar torque, the average peak power and the total positive work performed by the Chopart joint were significantly lower than those in the control group, and a trend towards reduced power absorption and total negative work were also observed on the healthy side of the patient’s foot, which seemed to reveal a symmetrical avoidance strategy. Suciu et al. (2016) evaluated the recovery effect of rehabilitation therapy. They analysed gait variables and functional outcomes at the time of weight loss (T1) and 12 weeks after the exercise rehabilitation programme (T2). The results showed that all spatial and temporal parameters were lower in T1 than in T2. Improvements were observed at T2, and the Olerud-Molander Ankle Scale (OMAS) score was also significantly improved. Therefore, the authors emphasized the importance of rehabilitation exercises in restoring patient movement.

### 4.7 Ankle osteoarthritis

Degenerative joint disease (DJD) of the ankle is a chronic disease associated with severe pain and dysfunction. Compared with primary ankle osteoarthritis, secondary osteoarthritis caused by traumatic injury and ankle biomechanical abnormalities (CAIs) is more common (Valderrabano et al., 2009). Joint disharmony caused by these pathological changes causes chronic cartilage overload and irreversible damage to the tibiotalar articular cartilage and eventually leads to narrowing and disappearance of the joint space, resulting in pain and deformity. Canseco et al. (2018) research team also confirmed that the dynamic ROMs of the tibia, hindfoot, forefoot and hallux of DJD patients decreased, the segmental position changed, and the foot as a whole flattened the arch, which reduced plantar fascia pretension, resulting in insufficient propulsion at the onset of the swing phase, as well as a significant decrease in the peak force and pressure values of the entire foot (especially in the hindfoot and toe regions). In the late stage of OA, the maximum force and peak pressure of the whole foot significantly decrease (especially in the hindfoot and toe areas), and the contact area with the ground also significantly decreases, which is interpreted as an “escape strategy” adopted by patients to avoid pain, which leads to insufficient support when entering the wobble phase (Horisberger et al., 2009). The combination of these factors leads to a decrease in spatiotemporal parameters and stability.

Total ankle replacement (TAR) and ankle arthrodesis (AA) are standard treatments for end-stage ankle osteoarthritis. Both procedures can effectively relieve pain caused by ankle arthritis. However, with the continuous improvement in quality of life and concerns about complications such as adjacent arthritis, an increasing number of people tend to choose TAR. Compared with those after AA, patients after TAR have a faster walking speed and greater ROM of the forefoot and hindfoot in the sagittal position (Seo et al., 2017). When completing more difficult tasks, such as climbing stairs, greater joint mobility allows for greater production of power and angular velocity, which makes the gait of TAR patients closer to normal, resulting in better exercise capability, full enjoyment of different exercise modalities, and improved quality of life (Sanders et al., 2021). Fritz et al. (2022) also showed that TAR surgery could increase the sagittal ROM of the ankle–joint complex, reduce the external rotation of the tibia, increase the external rotation of the hindfoot, relieve pain and improve walking function. However, the author also noted that although the outcome and gait ability of the study participants improved, there were significant differences in all the parameters in the healthy control group, indicating that TRA could not completely restore gait to normal. In the future, further research is needed on the prosthesis design, surgical methods, and postoperative rehabilitation of TAR to restore normal kinematic function.

## 5 New gait analysis technologies

Traditional gait analysis involves the use of computer vision technology; through the capture of fluorescent markers on the surface, the skin is subjected to three-dimensional modelling to determine the posture of the human body. However, when motions such as gait are performed, soft tissue displacement causes relative motion between a marker and its corresponding bone, thereby affecting the derived kinematics, especially in foot movement capture; because the distance between the markers is very small, this motion will lead to a relatively large angular error (Schallig et al., 2021).

Biplane fluoroscopy (BF) is a method for measuring 3D joint kinematics with high accuracy using two common bead-tracking techniques (fluoroscopic radiostereometric analysis [RSA]) or 2D-3D model-based registration (Harman et al., 2012; Akbari-Shandiz et al., 2018). These methods create a 3D model of each bone from imaging parameters to visualize the relative motion of a specific bone, avoiding the shortcomings of optical motion capture. Balsdon compared the three foot-types by quantifying the medial longitudinal arch (MLA) as an angular value and comparing the changes in MLA during static and dynamic processes. The results showed that the pes planus and pes cavus had the smallest and largest average MLA angles, respectively, and that the (static) barefoot MLA angle was less than the (dynamic) angle (Balsdon et al., 2016). Phan compared the relative motion of the joint surfaces of the planus foot and normal foot (Phan et al., 2021). The average relative speed on the articular surface of the tibiotalar, subtalar and calcaneocuboid joints was significantly greater in flatfoot patients. Compared with those of normal individuals, the plantar foot of patients exhibited increased movement towards plantar flexion in the tibiotalar joint and eversion and external rotations in the talonavicular joint during standing. The ROM of inversion/eversion rotations and internal/external rotations in the tibiotalar joint was significantly greater, demonstrating high instability. Another study on ankle stability compared the mobility of the tibiotalar and subtalar joints in FAI patients, LAS patients and normal subjects during the stance phase of walking, and the results showed that both FAI and LAS patients exhibited hypermobility of the tibiotalar joints. In contrast to LAS patients, despite having no apparent joint laxity under static conditions, FAI patients also showed significantly greater hypermobility of subtalar joints than healthy controls did, which confirmed that stabilization of the subtalar joints is needed for the treatment of patients with FAI (Cao et al., 2019). In an osteoarthritis study (Lenz et al., 2022), Lenz observed no significant kinematic differences in the tibiotalar joints during walking compared to those in the control group of post-TAR patients, with only a slight loss of joint mobility in the subtalar joints but still with symmetric kinematics during walking and double heel-rise activities. This provides a reference for the choice of end-stage OA treatment.

Although the BF can directly track bone movement, it also has several limitations. The large space occupation of the equipment, the high requirements for the surrounding environment, the complex and changeable professional knowledge of the equipment software, the limited collection environment and the excessive radiation dose are still the main factors that hinder the development of double-head fluoroscopy technology. With the development of technology, scholars are paying increasing attention to multisystem and multimodal sensor fusion methods. These methods can analyse the complexity and variability of gait by integrating the data of multiple sensors. Multimodal sensor fusion also allows simultaneous monitoring of various physiological parameters during exercise, such as various gait indices (spatiotemporal, kinematic and kinetic), sEMG signals, or tendon-ligament movement combined with ultrasound (Maeda et al., 2022). Fusion analysis of these indicators can reveal potential health conditions and disease causes, improve the accuracy and personalization of diagnosis, improve the prediction of disease, evaluate treatment efficacy, and guide rehabilitation.

## 6 Conclusion and foresight

The human body structure and the lower-limb kinetic chain have certain continuity and integrity; therefore, when there is a problem in a certain body part, there are often compensatory changes in adjacent joints. Numerous studies have noted the impact of hip and knee joint diseases on the ankle joint (Levinger et al., 2010b; Gao et al., 2016; Xie et al., 2019). Therefore, in the treatment of lower limb-related diseases, in addition to the treatment of clinical manifestations, it is also necessary to trace the source of the primary disease to fundamentally solve the symptoms. As a novel semi-automated dynamic assessment tool with the advantage of objectivity and precision in the diagnosis and assessment of abnormal gait compared to traditional clinical assessment, 3D gait analysis technology is now widely used in a number of disciplines to help guide diagnosis, assist in the development of surgical protocols, the assessment of treatment outcomes, and the guidance of rehabilitation training for disease, by capturing quantitative information about human movement. Currently, the biggest obstacle to incorporating this analysis remains the high cost (laboratory construction, time spent learning the technology and research testing). Increasing the convenience of gait analysis tools (especially those that can be used in an outpatient setting) without requiring patient access to the laboratory, reducing the cost of learning gait techniques, or shortening the time spent on laboratory testing will increase their utility as a clinical assessment tool, which will promote greater understanding of pathology and ultimately lead to better outcomes for patients.

## References

[B1] Akbari-Shandiz M. Mozingo J. D. Holmes D. R. Iii Zhao K. D. (2018). An interpolation technique to enable accurate three-dimensional joint kinematic analyses using asynchronous biplane fluoroscopy. Med. Eng. Phys. 60, 109–116. 10.1016/j.medengphy.2018.07.007 30098937

[B2] Alam U. Riley D. R. Jugdey R. S. Azmi S. Rajbhandari S. D'Août K. (2017). Diabetic neuropathy and gait: a review. Diabetes Ther. 8 (6), 1253–1264. 10.1007/s13300-017-0295-y 28864841 PMC5688977

[B3] Ansar A. Rahman A. E. Romero L. Haider M. R. Rahman M. M. Moinuddin M. (2018). Systematic review and meta-analysis of global birth prevalence of clubfoot: a study protocol. BMJ Open 8 (3), e019246. 10.1136/bmjopen-2017-019246 PMC5855200 29511012

[B4] Arnold J. B. Mackintosh S. Jones S. Thewlis D. (2013). Repeatability of stance phase kinematics from a multi-segment foot model in people aged 50 years and older. Gait Posture 38 (2), 349–351. 10.1016/j.gaitpost.2012.11.010 23219780

[B5] Baker R. Esquenazi A. Benedetti M. G. Desloovere K. (2016). Gait analysis: clinical facts. Eur. J. Phys. Rehabil. Med. 52 (4), 560–574. 27618499

[B6] Baker R. McGinley J. L. Schwartz M. H. Beynon S. Rozumalski A. Graham H. K. (2009). The gait profile score and movement analysis profile. Gait Posture 30 (3), 265–269. 10.1016/j.gaitpost.2009.05.020 19632117

[B7] Bakker J. P. De Groot I. J. Beelen A. Lankhorst G. J. (2002). Predictive factors of cessation of ambulation in patients with Duchenne muscular dystrophy. Am. J. Phys. Med. Rehabil. 81 (12), 906–912. 10.1097/00002060-200212000-00004 12447089

[B8] Ballas R. Edouard P. Philippot R. Farizon F. Delangle F. Peyrot N. (2016). Ground-reactive forces after hallux valgus surgery: comparison of Scarf osteotomy and arthrodesis of the first metatarsophalangeal joint. Bone Jt. J. 98-B (5), 641–646. 10.1302/0301-620X.98B5.36406 27143735

[B9] Balsdon M. E. Bushey K. M. Dombroski C. E. LeBel M. E. Jenkyn T. R. (2016). Medial longitudinal arch angle presents significant differences between foot types: a biplane fluoroscopy study. J. Biomech. Eng. 138(10). 10.1115/1.4034463 27548905

[B10] Bergerault F. Fournier J. Bonnard C. (2013). Idiopathic congenital clubfoot: initial treatment. Orthop. Traumatol. Surg. Res. 99 (1 Suppl. l), S150–S159. 10.1016/j.otsr.2012.11.001 23347754

[B11] Beyaert C. Haumont T. Paysant J. Lascombes P. Andre J. M. (2003). The effect of inturning of the foot on knee kinematics and kinetics in children with treated idiopathic clubfoot. Clin. Biomech. (Bristol, Avon) 18 (7), 670–676. 10.1016/s0268-0033(03)00114-1 12880715

[B12] Bigouette J. Simon J. Liu K. Docherty C. L. (2016). Altered vertical ground reaction forces in participants with chronic ankle instability while running. J. Athl. Train. 51 (9), 682–687. 10.4085/1062-6050-51.11.11 27813684 PMC5139784

[B13] Bombelli F. Stojkovic T. Dubourg O. Echaniz-Laguna A. Tardieu S. Larcher K. (2014). Charcot-Marie-Tooth disease type 2A: from typical to rare phenotypic and genotypic features. JAMA Neurol. 71 (8), 1036–1042. 10.1001/jamaneurol.2014.629 24957169

[B14] Böpple J. C. Tanner M. Campos S. Fischer C. Müller S. Wolf S. I. (2022). Short-term results of gait analysis with the Heidelberg foot measurement method and functional outcome after operative treatment of ankle fractures. J. Foot Ankle Res. 15 (1), 2. 10.1186/s13047-021-00505-4 34998420 PMC8742407

[B15] Brodsky J. W. Polo F. E. Coleman S. C. Bruck N. (2011). Changes in gait following the scandinavian total ankle replacement. J. Bone Jt. Surg. Am. 93 (20), 1890–1896. 10.2106/JBJS.J.00347 22012526

[B16] Buldt A. K. Forghany S. Landorf K. B. Levinger P. Murley G. S. Menz H. B. (2018b). Foot posture is associated with plantar pressure during gait: a comparison of normal, planus and cavus feet. Gait Posture 62, 235–240. 10.1016/j.gaitpost.2018.03.005 29573666

[B17] Buldt A. K. Forghany S. Landorf K. B. Murley G. S. Levinger P. Menz H. B. (2018a). Centre of pressure characteristics in normal, planus and cavus feet. J. Foot Ankle Res 11 (3), 10.1186/s13047-018-0245-6 PMC5800032 29441131

[B18] Buldt A. K. Levinger P. Murley G. S. Menz H. B. Nester C. J. Landorf K. B. (2015). Foot posture is associated with kinematics of the foot during gait: a comparison of normal, planus and cavus feet. Gait posture 42 (1), 42–48. 10.1016/j.gaitpost.2015.03.004 25819716

[B19] Bus S. A. Maas M. Michels R. P. Levi M. (2009). Role of intrinsic muscle atrophy in the etiology of claw toe deformity in diabetic neuropathy may not be as straightforward as widely believed. Diabetes Care 32 (6), 1063–1067. 10.2337/dc08-2174 19279305 PMC2681028

[B20] Canseco K. Kruger K. M. Fritz J. M. Konop K. A. Tarima S. Marks R. M. (2018). Distribution of segmental foot kinematics in patients with degenerative joint disease of the ankle. J. Orthop. Res. 36 (6), 1739–1746. 10.1002/jor.23807 29139570

[B21] Cao S. Wang C. Zhang G. Ma X. Wang X. Huang J. (2019). *In vivo* kinematics of functional ankle instability patients during the stance phase of walking. Gait Posture 73, 262–268. 10.1016/j.gaitpost.2019.07.377 31382233

[B22] Choi J. K. Cha E. J. Kim K. A. Won Y. Kim J. J. (2015). Effects of custom-made insoles on idiopathic pes cavus foot during walking. Biomed. Mater Eng. 26 (Suppl. 1), S705–S715. 10.3233/BME-151362 26406066

[B23] Cornwall M. W. McPoil T. G. (2000). Velocity of the center of pressure during walking. J. Am. Podiatr. Med. Assoc. 90 (7), 334–338. 10.7547/87507315-90-7-334 10933001

[B24] Coughlin M. J. Shurnas P. S. (2003). Hallux rigidus: demographics, etiology, and radiographic assessment. Foot Ankle Int. 24 (10), 731–743. 10.1177/107110070302401002 14587987

[B25] Court-Brown C. M. Caesar B. (2006). Epidemiology of adult fractures: a review. Injury 37 (8), 691–697. 10.1016/j.injury.2006.04.130 16814787

[B26] Crosbie J. Burns J. Ouvrier R. A. (2008). Pressure characteristics in painful pes cavus feet resulting from Charcot-Marie-Tooth disease. Gait Posture 28 (4), 545–551. 10.1016/j.gaitpost.2008.03.011 18456499

[B27] Debi R. Elbaz A. Mor A. Kahn G. Peskin B. Beer Y. (2017). Knee osteoarthritis, degenerative meniscal lesion and osteonecrosis of the knee: can a simple gait test direct us to a better clinical diagnosis. Orthop. Traumatol. Surg. Res. 103 (4), 603–608. 10.1016/j.otsr.2017.02.006 28330798

[B28] De Doncker E. Kowalski C. (1979). Cinesiologie et re education du pied. Paris: Masson Press.

[B29] Delahunt E. Monaghan K. Caulfield B. (2006). Altered neuromuscular control and ankle joint kinematics during walking in subjects with functional instability of the ankle joint. Am. J. Sports Med. 34 (12), 1970–1976. 10.1177/0363546506290989 16926342

[B30] De Mits S. Segers V. Woodburn J. Elewaut D. De Clercq D. Roosen P. (2012). A clinically applicable six-segmented foot model. J. Orthop. Res. 30 (4), 655–661. 10.1002/jor.21570 22021089

[B31] Deschamps K. Birch I. Desloovere K. Matricali G. A. (2010). The impact of hallux valgus on foot kinematics: a cross-sectional, comparative study. Gait Posture 32 (1), 102–106. 10.1016/j.gaitpost.2010.03.017 20451392

[B32] Deschamps K. Wouters J. Staes F. Vanstraelen E. Matricali G. A. Wuite S. (2022). Evidence for symmetrically reduced foot mechanics and energetics in patients after trimalleolar fracture repair: a cross-sectional study. Gait Posture 97, 13–20. 10.1016/j.gaitpost.2022.07.007 35849967

[B33] Doherty C. Bleakley C. Hertel J. Caulfield B. Ryan J. Delahunt E. (2015). Lower extremity function during gait in participants with first time acute lateral ankle sprain compared to controls. J. Electromyogr. Kinesiol 25 (1), 182–192. 10.1016/j.jelekin.2014.09.004 25443172

[B34] Egol K. A. Tejwani N. C. Walsh M. G. Capla E. L. Koval K. J. (2006). Predictors of short-term functional outcome following ankle fracture surgery. Tj. Bone Jt. Surg. Am. 88 (5), 974–979. 10.2106/JBJS.E.00343 16651571

[B35] Elsoe R. Ostgaard S. E. Larsen P. (2018). Population-based epidemiology of 9767 ankle fractures. Foot Ankle Surg. 24 (1), 34–39. 10.1016/j.fas.2016.11.002 29413771

[B36] Feger M. A. Hart J. M. Saliba S. Abel M. F. Hertel J. (2018). Gait training for chronic ankle instability improves neuromechanics during walking. J. Orthop. Res. 36 (1), 515–524. 10.1002/jor.23639 28653780

[B37] Fernández-Gorgojo M. Salas-Gómez D. Sánchez-Juan P. Laguna-Bercero E. Pérez-Núñez M. I. (2023). Analysis of dynamic plantar pressure and influence of clinical-functional measures on their performance in subjects with bimalleolar ankle fracture at 6 and 12 Months post-surgery. Sensors (Basel) 23(8):3975. 10.3390/s23083975 37112316 PMC10142754

[B38] Fernández-Seguín L. M. Diaz Mancha J. A. Sánchez Rodríguez R. Escamilla Martínez E. Gómez Martín B. Ramos Ortega J. (2014). Comparison of plantar pressures and contact area between normal and cavus foot. Gait Posture 39 (2), 789–792. 10.1016/j.gaitpost.2013.10.018 24220205

[B39] Fritz J. M. Canseco K. Konop K. A. Kruger K. M. Tarima S. Long J. T. (2022). Multi-segment foot kinematics during gait following ankle arthroplasty. J. Orthop. Res. 40 (3), 685–694. 10.1002/jor.25062 33913547

[B40] Galica A. M. Hagedorn T. J. Dufour A. B. Riskowski J. L. Hillstrom H. J. Casey V. A. (2013). Hallux valgus and plantar pressure loading: the Framingham foot study. J. Foot Ankle Res. 6 (1), 42. 10.1186/1757-1146-6-42 24138804 PMC3819471

[B41] Ganesan B. Luximon A. Al-Jumaily A. Balasankar S. K. Naik G. R. (2017). Ponseti method in the management of clubfoot under 2 years of age: a systematic review. PLoS One 12 (6), e0178299. 10.1371/journal.pone.0178299 28632733 PMC5478104

[B42] Gao F. Ma J. Sun W. Guo W. Li Z. Wang W. (2016). The influence of knee malalignment on the ankle alignment in varus and valgus gonarthrosis based on radiographic measurement. Eur. J. Radiol. 85 (1), 228–232. 10.1016/j.ejrad.2015.11.021 26724670

[B43] Geng X. Huang D. Wang X. Zhang C. Huang J. Ma X. (2017). Loading pattern of postoperative hallux valgus feet with and without transfer metatarsalgia: a case control study. J. Orthop. Surg. Res. 12 (1), 120. 10.1186/s13018-017-0622-z 28743301 PMC5526287

[B44] Goost H. Wimmer M. D. Barg A. Kabir K. Valderrabano V. Burger C. (2014). Fractures of the ankle joint: investigation and treatment options. Dtsch. Arztebl Int. 111 (21), 377–388. 10.3238/arztebl.2014.0377 24939377 PMC4075279

[B45] Grin L. van der Steen M. C. Wijnands S. D. N. van Oorschot L. Besselaar A. T. Vanwanseele B. (2021). Forefoot adduction and forefoot supination as kinematic indicators of relapse clubfoot. Gait Posture 90, 415–421. 10.1016/j.gaitpost.2021.09.185 34583148

[B46] Grin L. van Oorschot L. Vanwanseele B. Wijnands S. D. N. Kars H. J. J. C. Besselaar A. T. (2023). Kinematic gait impairments in children with clubfeet treated by the Ponseti method: a systematic review and meta-analysis. Child. (Basel) 10 (5), 785. 10.3390/children10050785 PMC10217440 37238333

[B47] Grin L. Wijnands S. Besselaar A. van Oorschot L. Vanwanseele B. van der Steen M. (2022). The relation between clinical and objective gait scores in clubfoot patients with and without a relapse. Gait Posture 97, 210–215. 10.1016/j.gaitpost.2022.07.261 35995000

[B48] Guillebastre B. Calmels P. Rougier P. (2013). Effects of muscular deficiency on postural and gait capacities in patients with Charcot-Marie-Tooth disease. J. Rehabil. Med. 45 (3), 314–317. 10.2340/16501977-1113 23412436

[B49] Halabchi F. Hassabi M. (2020). Acute ankle sprain in athletes: clinical aspects and algorithmic approach. World J. Orthop. 11 (12), 534–558. 10.5312/wjo.v11.i12.534 33362991 PMC7745493

[B50] Harman M. K. Banks S. A. Kirschner S. Lützner J. (2012). Prosthesis alignment affects axial rotation motion after total knee replacement: a prospective *in vivo* study combining computed tomography and fluoroscopic evaluations. BMC Musculoskelet. Disord. 13:206. 10.1186/1471-2474-13-206 23088451 PMC3575259

[B51] Hecht G. G. Van Rysselberghe N. L. Young J. L. Gardner M. J. (2022). Gait analysis in orthopaedic surgery: history, limitations, and future directions. J. Am. Acad. Orthop. Surg. 30 (21), e1366–e1373. 10.5435/JAAOS-D-21-00785 36026713

[B52] Henley J. Richards J. Hudson D. Chruch C. Coleman S. Kerstetter L. (2008). “Reliability of a clinically practical multi-segment foot marker set,” in Foot ankle motion anal. Clin. Treat. Technol. Editors Harris G. Smith P. Marks R. (Boca Raton, FL: CRC Press), 445–464.

[B53] Horisberger M. Hintermann B. Valderrabano V. (2009). Alterations of plantar pressure distribution in posttraumatic end-stage ankle osteoarthritis. Clin. Biomech. (Bristol, Avon) 24 (3), 303–307. 10.1016/j.clinbiomech.2008.12.005 19150745

[B54] Kadaba M. P. Ramakrishnan H. K. Wootten M. E. (1990). Measurement of lower extremity kinematics during level walking. J. Orthop. Res. 8 (3), 383–392. 10.1002/jor.1100080310 2324857

[B55] Kalkum E. van Drongelen S. Mussler J. Wolf S. I. Kuni B. (2016). A marker placement laser device for improving repeatability in 3D-foot motion analysis. Gait Posture 44, 227–230. 10.1016/j.gaitpost.2015.12.024 27004663

[B56] Kerr C. M. Stebbins J. Theologis T. Zavatsky A. B. (2015). Static postural differences between neutral and flat feet in children with and without symptoms. Clin. Biomech. (Bristol, Avon) 30 (3), 314–317. 10.1016/j.clinbiomech.2015.02.007 25721676

[B57] Kerr C. M. Zavatsky A. B. Theologis T. Stebbins J. (2019). Kinematic differences between neutral and flat feet with and without symptoms as measured by the Oxford foot model. Gait Posture 67, 213–218. 10.1016/j.gaitpost.2018.10.015 30368208

[B58] Kidder S. M. Abuzzahab F. S. Jr Harris G. F. Johnson J. E. (1996). A system for the analysis of foot and ankle kinematics during gait. IEEE Trans. Rehabil. Eng. 4 (1), 25–32. 10.1109/86.486054 8798069

[B59] Kim E. J. Shin H. S. Takatori N. Yoo H. J. Cho Y. J. Yoo W. J. (2020). Inter-segmental foot kinematics during gait in elderly females according to the severity of hallux valgus. J. Orthop. Res. 38 (11), 2409–2418. 10.1002/jor.24657 32162717

[B60] Koldenhoven R. M. Feger M. A. Fraser J. J. Saliba S. Hertel J. (2016). Surface electromyography and plantar pressure during walking in young adults with chronic ankle instability. Knee Surg. Sports Traumatol. Arthrosc. 24 (4), 1060–1070. 10.1007/s00167-016-4015-3 26856315

[B61] Kruger K. M. Graf A. Flanagan A. McHenry B. D. Altiok H. Smith P. A. (2019). Segmental foot and ankle kinematic differences between rectus, planus, and cavus foot types. J. Biomech. 94, 180–186. 10.1016/j.jbiomech.2019.07.032 31420153

[B62] Kuo A. D. Donelan J. M. (2010). Dynamic principles of gait and their clinical implications. Phys. Ther. 90 (2), 157–174. 10.2522/ptj.20090125 20023002 PMC2816028

[B63] Kwon O. Y. Tuttle L. J. Johnson J. E. Mueller M. J. (2009). Muscle imbalance and reduced ankle joint motion in people with hammer toe deformity. Clin. Biomech. (Bristol, Avon) 24 (8), 670–675. 10.1016/j.clinbiomech.2009.05.010 19535185 PMC2751588

[B64] Kwon Y. U. Harrison K. Kweon S. J. Williams D. S. B. 3rd (2020). Ankle coordination in chronic ankle instability, coper, and control groups in running. Med. Sci. Sports Exerc 52 (3), 663–672. 10.1249/MSS.0000000000002170 31652242

[B65] Leardini A. Benedetti M. G. Berti L. Bettinelli D. Nativo R. Giannini S. (2007). Rear-foot, mid-foot and fore-foot motion during the stance phase of gait. Gait Posture 25 (3), 453–462. 10.1016/j.gaitpost.2006.05.017 16965916

[B66] Lenz A. L. Lisonbee R. J. Peterson A. C. Roach K. E. Foreman K. B. Barg A. (2022). Total ankle replacement provides symmetrical postoperative kinematics: a biplane fluoroscopy imaging study. Foot Ankle Int. 43 (6), 818–829. 10.1177/10711007221078001 35293257 PMC9980879

[B67] Levinger P. Menz H. B. Fotoohabadi M. R. Feller J. A. Bartlett J. R. abd Bergman N. R. (2010b). Foot posture in people with medial compartment knee osteoarthritis. J. Foot Ankle Res. 3, 29. 10.1186/1757-1146-3-29 21162748 PMC3020154

[B68] Levinger P. Murley G. S. Barton C. J. Cotchett M. P. McSweeney S. R. Menz H. B. (2010a). A comparison of foot kinematics in people with normal- and flat-arched feet using the Oxford Foot Model. Gait Posture 32 (4), 519–523. 10.1016/j.gaitpost.2010.07.013 20696579

[B69] Li J. Xun F. Li Y. Liu Y. Xu H. Canavese F. (2022). Three-dimensional gait analysis in children with recurrent idiopathic clubfoot undergoing complete tibialis anterior tendon transfer. J. Pediatr. Orthop. B 31 (4), 397–406. 10.1097/BPB.0000000000000941 34908029

[B70] Liu Y. B. Jiang S. Y. Zhao L. Yu Y. Zhao D. H. (2020). Can repeated Ponseti management for relapsed clubfeet produce the outcome comparable with the case without relapse? A clinical study in term of gait analysis. J. Pediatr. Orthop. 40 (1), 29–35. 10.1097/BPO.0000000000001071 31815859

[B71] Long J. T. Eastwood D. C. Graf A. R. Smith P. A. Harris G. F. (2010). Repeatability and sources of variability in multi-center assessment of segmental foot kinematics in normal adults. Gait Posture 31 (1), 32–36. 10.1016/j.gaitpost.2009.08.240 19775894

[B72] Lööf E. Andriesse H. André M. Böhm S. Broström E. W. (2016). Gait in 5-year-old children with idiopathic clubfoot: a cohort study of 59 children, focusing on foot involvement and the contralateral foot. Acta Orthop. 87 (5), 522–528. 10.1080/17453674.2016.1202013 27331243 PMC5016913

[B73] MacWilliams B. A. Cowley M. Nicholson D. E. (2003). Foot kinematics and kinetics during adolescent gait. Gait Posture 17 (3), 214–224. 10.1016/s0966-6362(02)00103-0 12770635

[B74] Maeda N. Ikuta Y. Tashiro T. Arima S. Morikawa M. Kaneda K. (2022). Quantitative evaluation of the vertical mobility of the first tarsometatarsal joint during stance phase of gait. Sci. Rep. 12(1):9246. 10.1038/s41598-022-13425-5 35655091 PMC9163033

[B75] Mannoni A. Briganti M. P. Di Bari M. Ferrucci L. Costanzo S. Serni U. (2003). Epidemiological profile of symptomatic osteoarthritis in older adults: a population based study in Dicomano, Italy. Ann. Rheum. Dis. 62 (6), 576–578. 10.1136/ard.62.6.576 12759299 PMC1754567

[B76] Mcdonald S. W. Tavener G. (1999). Pronation and supination of the foot: confused terminology. Foot 9 (1), 6–11. 10.1054/foot.1999.0502

[B77] Menz H. B. Auhl M. Tan J. M. Buldt A. K. Munteanu S. E. (2018). Centre of pressure characteristics during walking in individuals with and without first metatarsophalangeal joint osteoarthritis. Gait Posture 63, 91–96. 10.1016/j.gaitpost.2018.04.032 29727777

[B78] Mindler G. T. Kranzl A. Lipkowski C. A. Ganger R. Radler C. (2014). Results of gait analysis including the Oxford foot model in children with clubfoot treated with the Ponseti method. J. Bone Jt. Surg. Am. 96 (19), 1593–1599. 10.2106/JBJS.M.01603 25274784

[B79] Mindler G. T. Kranzl A. Radler C. (2020). Normalization of forefoot supination after tibialis anterior tendon transfer for dynamic clubfoot recurrence. J. Pediatr. Orthop. 40 (8), 418–424. 10.1097/BPO.0000000000001542 32205682

[B80] Moerenhout K. Chopra S. Crevoisier X. (2019). Outcome of the modified Lapidus procedure for hallux valgus deformity during the first year following surgery: a prospective clinical and gait analysis study. Clin. Biomech. (Bristol, Avon) 61, 205–210. 10.1016/j.clinbiomech.2018.12.017 30594769

[B81] Ng S. S. Hui-Chan C. W. (2012). Contribution of ankle dorsiflexor strength to walking endurance in people with spastic hemiplegia after stroke. Arch. Phys. Med. Rehabil. 93 (6), 1046–1051. 10.1016/j.apmr.2011.12.016 22440486

[B82] Nix S. Smith M. Vicenzino B. (2010). Prevalence of hallux valgus in the general population: a systematic review and meta-analysis. J. Foot Ankle Res. 3, 21. 10.1186/1757-1146-3-21 20868524 PMC2955707

[B83] Novak A. C. Mayich D. J. Perry S. D. Daniels T. R. Brodsky J. W. (2014). Gait analysis for foot and ankle surgeons-- topical review, part 2: approaches to multisegment modeling of the foot. Foot Ankle Int. 35 (2), 178–191. 10.1177/1071100713511435 24334310

[B84] Oosterwaal M. Telfer S. Tørholm S. Carbes S. van Rhijn L. W. Macduff R. (2011). Generation of subject-specific, dynamic, multisegment ankle and foot models to improve orthotic design: a feasibility study. BMC Musculoskelet. Disord. 12, 256. 10.1186/1471-2474-12-256 22074482 PMC3234203

[B85] Peng Y. Wong D. W. Wang Y. Chen T. L. Tan Q. Chen Z. (2020). Immediate effects of medially posted insoles on lower limb joint contact forces in adult acquired flatfoot: a pilot study. Int. J. Environ. Res. Public Health 17 (7), 2226. 10.3390/ijerph17072226 32224985 PMC7178021

[B86] Phan C. B. Lee K. M. Kwon S. S. Koo S. (2021). Kinematic instability in the joints of flatfoot subjects during walking: a biplanar fluoroscopic study. J. Biomech. 127, 110681. 10.1016/j.jbiomech.2021.110681 34438290

[B87] Ponseti I. V. Zhivkov M. Davis N. Sinclair M. Dobbs M. B. Morcuende J. A. (2006). Treatment of the complex idiopathic clubfoot. Clin. Orthop. Relat. Res. 451, 171–176. 10.1097/01.blo.0000224062.39990.48 16788408

[B88] Powell D. W. Williams D. S. Butler R. J. (2013). A comparison of two multisegment foot models in high-and low-arched athletes. J. Am. Podiatr. Med. Assoc. 103 (2), 99–105. 10.7547/1030099 23536499

[B89] Riskowski J. L. Dufour A. B. Hagedorn T. J. Hillstrom H. J. Casey V. A. Hannan M. T. (2013). Associations of foot posture and function to lower extremity pain: results from a population-based foot study. Arthritis Care Res. Hob. 65 (11), 1804–1812. 10.1002/acr.22049 PMC4039193 24591410

[B90] Sanchis-Sales E. Rodríguez-Cervantes P. J. Sancho-Bru J. L. (2019). Kinematics reduction applied to the comparison of highly-pronated, normal and highly-supinated feet during walking. Gait Posture 68, 269–273. 10.1016/j.gaitpost.2018.12.010 30551052

[B91] Sanders A. E. Kraszewski A. P. Ellis S. J. Queen R. Backus S. I. Hillstrom H. (2021). Differences in gait and stair ascent after total ankle arthroplasty and ankle arthrodesis. Foot Ankle Int. 42 (3), 347–355. 10.1177/1071100720965144 33198507

[B92] Saraswat P. MacWilliams B. A. Davis R. B. (2012). A multi-segment foot model based on anatomically registered technical coordinate systems: method repeatability in pediatric feet. Gait Posture 35 (4), 547–555. 10.1016/j.gaitpost.2011.11.022 22192872

[B93] Saraswat P. MacWilliams B. A. Davis R. B. D'Astous J. L. (2013). A multi-segment foot model based on anatomically registered technical coordinate systems: method repeatability and sensitivity in pediatric planovalgus feet. Gait Posture 37 (1), 121–125. 10.1016/j.gaitpost.2012.06.023 22858244

[B94] Schallig W. Streekstra G. J. Hulshof C. M. Kleipool R. P. Dobbe J. G. G. Maas M. (2021). The influence of soft tissue artifacts on multi-segment foot kinematics. J. Biomech. 120, 110359. 10.1016/j.jbiomech.2021.110359 33730563

[B95] Schallig W. van den Noort J. C. Piening M. Streekstra G. J. Maas M. van der Krogt M. M. (2022). The Amsterdam Foot Model: a clinically informed multi-segment foot model developed to minimize measurement errors in foot kinematics. J. Foot Ankle Res. 15 (1), 46. 10.1186/s13047-022-00543-6 35668453 PMC9172122

[B96] Schelhaas R. Hajibozorgi M. Hortobágyi T. Hijmans J. M. Greve C. (2022). Conservative interventions to improve foot progression angle and clinical measures in orthopedic and neurological patients - a systematic review and meta-analysis. J. Biomech. 130, 110831. 10.1016/j.jbiomech.2021.110831 34741811

[B97] Schmidt R. Benesch S. Friemert B. Herbst A. Claes L. Gerngross H. (2005). Anatomical repair of lateral ligaments in patients with chronic ankle instability. Knee Surg. Sports Traumatol. Arthrosc. 13 (3), 231–237. 10.1007/s00167-004-0562-0 15824935

[B98] Schwartz M. H. Rozumalski A. (2008). The Gait Deviation Index: a new comprehensive index of gait pathology. Gait Posture 28 (3), 351–357. 10.1016/j.gaitpost.2008.05.001 18565753

[B99] Seo S. G. Kim E. J. Lee D. J. Bae K. J. Lee K. M. Lee D. Y. (2017). Comparison of multisegmental foot and ankle motion between total ankle replacement and ankle arthrodesis in adults. Foot Ankle Int. 38 (9), 1035–1044. 10.1177/1071100717709564 28587575

[B100] Seo S. G. Lee D. Y. Moon H. J. Kim S. J. Kim J. Lee K. M. (2014). Repeatability of a multi-segment foot model with a 15-marker set in healthy adults. J. Foot Ankle Res. 7, 24. 10.1186/1757-1146-7-24 24782914 PMC4004446

[B101] Shibuya N. Davis M. L. Jupiter D. C. (2014). Epidemiology of foot and ankle fractures in the United States: an analysis of the national trauma data bank (2007 to 2011). J. Foot Ankle Surg. 53 (5), 606–608. 10.1053/j.jfas.2014.03.011 24785202

[B102] Simon J. Doederlein L. McIntosh A. S. Metaxiotis D. Bock H. G. Wolf S. I. (2006). The Heidelberg foot measurement method: development, description and assessment. Gait Posture 23 (4), 411–424. 10.1016/j.gaitpost.2005.07.003 16157483

[B103] Suciu O. Onofrei R. R. Totorean A. D. Suciu S. C. Amaricai E. C. (2016). Gait analysis and functional outcomes after twelve-week rehabilitation in patients with surgically treated ankle fractures. Gait Posture 49, 184–189. 10.1016/j.gaitpost.2016.07.006 27434488

[B104] Sutherland D. H. (2001). The evolution of clinical gait analysis part l: kinesiological EMG. Gait Posture 14 (1), 61–70. 10.1016/s0966-6362(01)00100-x 11378426

[B105] Sutherland D. H. (2002). The evolution of clinical gait analysis. Gait Posture 16 (2), 159–179. 10.1016/s0966-6362(02)00004-8 12297257

[B106] Sutherland D. H. (2005). The evolution of clinical gait analysis part III--kinetics and energy assessment. Gait Posture 21 (4), 447–461. 10.1016/j.gaitpost.2004.07.008 15886135

[B107] Tantigate D. Ho G. Kirschenbaum J. Bäcker H. Asherman B. Freibott C. (2019). Timing of open reduction and internal fixation of ankle fractures. Foot Ankle Spec. 12 (5), 401–408. 10.1177/1938640018810419 30426777

[B108] Theologis T. Stebbins J. (2010). The use of gait analysis in the treatment of pediatric foot and ankle disorders. Foot Ankle Clin. 15 (2), 365–382. 10.1016/j.fcl.2010.02.002 20534362

[B109] Valderrabano V. Horisberger M. Russell I. Dougall H. Hintermann B. (2009). Etiology of ankle osteoarthritis. Clin. Orthop. Relat. Res. 467 (7), 1800–1806. 10.1007/s11999-008-0543-6 18830791 PMC2690733

[B110] van Hoeve S. Houben M. Verbruggen J. P. A. M. Willems P. Meijer K. Poeze M. (2019). Gait analysis related to functional outcome in patients operated for ankle fractures. J. Orthop. Res. 37 (7), 1658–1666. 10.1002/jor.24071 29920765 PMC6618247

[B111] van Rijn R. M. van Os A. G. Bernsen R. M. Luijsterburg P. A. Koes B. W. Bierma-Zeinstra S. M. (2008). What is the clinical course of acute ankle sprains? A systematic literature review. Am. J. Med. 121 (4), 324–331.e7. 10.1016/j.amjmed.2007.11.018 18374692

[B112] Walker M. Fan H. J. (1998). Relationship between foot pressure pattern and foot type. Foot Ankle Int. 19 (6), 379–383. 10.1177/107110079801900607 9677081

[B113] Wallander H. Hovelius L. Michaelsson K. (2006). Incidence of congenital clubfoot in Sweden. Acta Orthop. 77 (6), 847–852. 10.1080/17453670610013123 17260191

[B114] Waterman B. R. Owens B. D. Davey S. Zacchilli M. A. Belmont P. J. Jr (2010). The epidemiology of ankle sprains in the United States. J. Bone Jt. Surg. Am. 92 (13), 2279–2284. 10.2106/JBJS.I.01537 20926721

[B115] Winter D. A. (2009). Biomechanics and motor control of human movement. Fourth Edition.

[B116] Wright C. J. Arnold B. L. Coffey T. G. Pidcoe P. E. (2011). Repeatability of the modified Oxford foot model during gait in healthy adults. Gait Posture 33 (1), 108–112. 10.1016/j.gaitpost.2010.10.084 21081275

[B117] Xie K. Han X. Jiang X. Ai S. Dai K. Yu Z. (2019). The effect of varus knee deformities on the ankle alignment in patients with knee osteoarthritis. J. Orthop. Surg. Res. 14 (1), 134. 10.1186/s13018-019-1191-0 31092268 PMC6521394

[B118] Xu C. Wei J. Yan Y. B. Shang L. Yang X. J. Huang L. Y. (2018). Pedobarographic analysis following Ponseti treatment for unilateral neglected congenital clubfoot. Sci. Rep. 8 (1), 6270. 10.1038/s41598-018-24737-w 29674653 PMC5908870

[B119] Yoo H. J. Park H. S. Lee D. O. Kim S. H. Park G. Y. Cho T. J. (2022). Comparison of the kinematics, repeatability, and reproducibility of five different multi-segment foot models. J. Foot Ankle Res. 15 (1), 1. 10.1186/s13047-021-00508-1 34991669 PMC8734222

[B120] Zhao D. H. Rao W. W. Zhao L. Yang X. Liu J. L. Wu Z. K. (2016). Are incidence and severity of clubfoot related to the season of birth? World J. Pediatr. 12 (3), 360–363. 10.1007/s12519-016-0029-7 27351571

[B121] Zhu T. Wang Y. Tian F. Wang W. Zhong R. Zhai H. (2022). Clinical assessments and gait analysis for patients with Trimalleolar fractures in the early postoperative period. BMC Musculoskelet. Disord 23(1):663. 10.1186/s12891-022-05615-z 35820837 PMC9275242

[B122] Zionts L. E. Ebramzadeh E. Morgan R. D. Sangiorgio S. N. (2018). Sixty years on: Ponseti method for clubfoot treatment produces high satisfaction despite inherent tendency to relapse. J. Bone Jt. Surg. Am. 100 (9), 721–728. 10.2106/JBJS.17.01024 29715219

