# Supplementary material for: Three-dimensional gait analysis of orthopaedic common foot and ankle joint diseases
Source: Front Bioeng Biotechnol. 2024 Feb 22;12:1303035. doi: 10.3389/fbioe.2024.1303035 (PMC10919227; doi:10.3389/fbioe.2024.1303035)
Supplement: Supplementary file 1 [file Table1.DOCX]

**Table 1 Different multi-segment foot models**

| **References** | **Model** | **Number of Segments** | **Segments** | **Advantages** |
| --- | --- | --- | --- | --- |
| Kadaba MP，et al,1990 [14] | Modified Helen Hayes foot model | **1** | Shank (Tib-Fib)  Foot | Can provide information on the ankle kinematics and kinetics |
| Kidder SM, et al,1996 [15] | Milwaukee Foot Model (**MiFM**) | **4** | Shank (Tib-Fib)  Hindfoot\Forefoot\Hallux | Marker assumptions require radiographs for calibration  Proven reliability among different centers |
| J. Henley, J, et al,2011 [16] | Dupont Foot Model (**DFM**) | **4** | Shank (Tib-Fib)  Hindfoot\Forefoot\Hallux | Proven reliability among different centers  Availability of software for data analysis |
| Wright CJ, et al,2011 [17] | Modified Oxford Foot Model (**OFM**) | **4** | Shank (Tib-Fib)  Hindfoot\Forefoot\Hallux | Proven reliability among different centers  Availability of software for data analysis |
| Leardini A, et al,2007 [18] | Rizzoli Foot Model (**RFM**) | **4** | Shank (Tib-Fib)  Hindfoot\Midfoot\ Hallux | Includes midfoot marker points and focuses on hindfoot position on the coronal plane |
| Saraswat, P, et al, 2012  [19] | modified Shriners Hospital for Children Greenville foot model (**mSHCG**) | **4** | Shank (Tib-Fib)  Hindfoot\Forefoot\Hallux | reduced required anatomical marker alignment by minimizing the number of anatomical markers and critical alignment directions  appropriate for pediatric subjects |
| Schallig W, et al,2022 [20] | Amsterdam Foot Model（**AFM**） | **5/6** | Shank (Tib-Fib)  Hindfoot\Midfoot  Forefoot（optionally divided into a Medial and Lateral forefoot）\Hallux | As a clinically informed multisegmental foot model that minimizes kinematic measurement error, is not specific to a particular patient population or age, and can be used in a wide range of clinical applications and patient populations |
| De Mits S, et al,2012 [21] | Ghent foot model (**GFM**) | **6** | Shank (Tib-Fib)  Hindfoot\Midfoot\Medial forefoot \Lateral forefoot \Hallux | Allows for increasing resolution in foot biomechanics of the forefoot  Include hindfoot, midfoot, first ray, and hallux can be used to evaluate the windlass mechanism |
| MacWilliams BA, et al,2003 [22] | MacWilliams Model | **9** | Shank (Tib-Fib)  Talus/navicular/cuneiform  Cuboid \Calcaneus \Medial forefoot \Lateral forefoot \Medial toes \Lateral toes\Hallux \Talus | Further refinement of the foot's internal segments allows for the integration of more information on foot kinematics and dynamics- |
| Simon, J, et al, 2006 [23] | Heidelberg Foot Measurement Method (**HFMM**) | **-** | - | The mid and forefoot, the method does not incorporate a standard rigid body model, but applies a descriptive method to assess foot motion parameters that are relevant to the clinician. |
| Oosterwaal, M, et al, 2011 [24] | Glasgow-Maastricht foot model | **26** | A forward dynamic model  An inverse dynamic model | The model will contain all of the ligaments and muscles of the foot and ankle. the model will provide insight in function of the foot and leg muscles during gait. |

**Table 2 Gait analysis techniques for different foot and ankle joint diseases**

| **Disease** | **Model** | **Subjects** | **Gait Analysis Result** |  |
| --- | --- | --- | --- | --- |
| **Cavus**  **Foot** | Plantar pressure test | Pes cavus: n = 34, Neutral feet: n =34 | The pes cavus showed a significant reduction in their weight-bearing areaand significantly increased pressures under all zones of the forefoot except the fifth metatarsal. | [47] |
|  | MFM | Cavus Group: n = 11, Rectus Group: n = 11, Planus Group: n = 11. | The Cavus Group showed increased dorsiflexion and inversion in the hindfoot and increased plantarflexion, valgus, and adduction in the forefoot. The Planus Group had less dorsiflexion, more eversion, and more external rotation in the hindfoot as well as less plantarflexion and increased varus in the forefoot. | [49] |
|  | A five segment foot model and marker set | Normal: n = 37, Pes cavus: n = 30, Pes planus: n = 30 | 1.Changes in frontal and transverse plane angles of the hindfoot of the cavus group; 2.less motion of the midfoot in the sagittal and transverse planes during initial contact and midstance in the cavus group; 3.reduced midfoot frontal plane ROM during pre-swing in the planus group.. | [50] |
|  | Plantar foot pressure and sEMG | Pes cavus: n = 10 | The custom-made insoles dispersed pressure concentrated by the higher medial longitudinal arch and improved the efficient use of muscles | [51] |
| **Planus**  **Foot** | Plantar pressure test | Normal: n = 35, Pes cavus: n = 26, Pes planus: n = 31 | The largest differences were between the planus and cavus foot groups in forefoot pressure and force. The peak pressures at the 4th and 5th MTPJs in the planus foot group were lower, and displayed the largest effect sizes. | [53] |
|  | OFM | Asymptomatic neutral: n = 88, Asymptomatic mild flatfoot: n = 47, Asymptomatic flatfoot: n = 29, Symptomatic flatfoot: n = 30 | Hindfoot eversion was increased in children with asymptomatic and, to a greater extent, symptomatic flatfoot. The forefoot was significantly more abducted in the symptomatic and in the flat group. The forefoot was more supinated relative to the hindfoot in the flatfoot groups. | [55] |
|  | Plug-in gait (PIG) and OFM | Asymptomatic neutral: n = 98, Asymptomatic mild flatfoot: n = 47, Asymptomatic flatfoot: n = 29, Symptomatic flatfoot: n = 38 | The symptomatic flat feet showed significant differences from asymptomatic groups (most commonly the neutral feet) in terms of hip flexion, knee flexion and varus, hindfoot inversion-eversion, and forefoot abduction-adduction. | [56] |
|  | Modified PIG and MSK | Flatfoot: n=15 | The second peak patellofemoral contact force and the peak ankle contact force were significantly lower in the WSFO group. The foot orthosis significantly reduced the peak ankle eversion angle and ankle eversion moment; however, the peak knee adduction moment increased. | [58] |
| **Congenital Talipes Equinovarus** | Plantar pressure test | NCF who underwent Ponseti treatment: n = 22, Healthy children n=22 | In the present study, a higher proportion of the internal FPA was found in the affected clubfeet. The affected feet showed a significant increase in CA% and a higher PP in the M5 and MF zones. Internal foot progression angle and a load transfer from the medial forefoot and hindfoot to the lateral forefoot and midfoot were observed in the affected feet. | [67] |
|  | Extended Helen-Hayes model and OFM | The group of successfully treated clubfoot (the nonrelapse group), Relapse clubfoot (the relapse group). | Clubfoot patients with a relapse show lower total gait quality (GDI*) and lower clinical status defined by the CAP. Abnormal cFDI* was found in relapse patients, reflected by differences in corresponding variable scores. Moderate relationships were found for the subdomains of the CAP and total gait and foot quality in all clubfoot patients. | [70] |
|  | Extended Helen-Hayes model and OFM | Control group: n = 15,  Corrected group: n = 11, Relapse group: n = 11 | The relapse group showed significantly increased forefoot adduction in relation with the hindfoot and the tibia. this group showed increased forefoot supination in relation with the tibia during stance, whereas during swing increased forefoot supination in relation with the hindfoot was found in patients with relapse clubfoot. | [71] |
|  | Helen Hayes model | Dynamic supination (recurrent clubfoot): n = 17 | The postoperative step length，stride length, postoperative peak internal ankle rotation angle in the frontal plane, postoperative peak internal foot progression angle in the transverse plane and V-angle-S values were significantly smaller than their preoperative values. | [72] |
|  | Cleveland clinical model and OFM | Recurrent clubfoot: n = 17, Healthy childr: n = 25en: n = 18 | After TATT, forefoot supination in relation to the hindfoot and tibia was reduced during swing and at initial contact, the heel showed less dynamic varus and adduction movement, Maximum ankle dorsiflexion slightly increased. Maximum ankle power was reduced preoperatively and postoperatively compared with controls. | [73] |
|  | Helen Hayes model | Relapsed clubfeet: n = 17, Clubfeet without relapse: n = 28 | There was statistically significant difference in the parameters of foot length, stride length, and single limb support time (%gait cycle) between the 2 groups | [74] |
| **Hallux**  **Valgus** | Plantar pressure test | A population-based study | Participants with HV had lower hallucal loading and higher forces at lesser toes as well as higher MAI and lower CPEI values compared to the referent. Participants with HV and other FDs were also noted to have aberrant rearfoot forces and pressures. | [77] |
|  | OFM | Hallux valgus participants: n = 20, Symptom-free volunteers: n = 22 | In our HV population we found an increased dorsiflexion motion at the hallux during terminal stance. In both sub-phases of stance, the HV group showed increased eversion of the hindfoot, indicating a less stable foot. | [78] |
|  | DFM | Female symptomatic HV patients n = 58, female nonsymptomatic older volunteers n = 50 | For temporal parameters, gait speed and stride length were diminished according to the severity of HV deformity. Sagittal range of motion of hallux and hindfoot decreased significantly and loss of push‐off during the preswing phase was observed and forefoot adduction motion during terminal stance was decreasedin SHV group. | [79] |
|  | Pressure insoles and five 3-D inertial sensors connected with two data-loggers | Female patients with moderate to severe hallux valgus who underwent modified Lapidus procedure: n =15 | Three spatiotemporal, two kinematics, and seven plantar pressure parameters significantly improved between 6 months and 12 months postoperatively. Significant improvement in radiological and clinical outcome was reported at 6 and 12 months. | [80] |
|  | Plantar pressure test | Consecutive feet with postoperative transfer metatarsalgia: n =30, Feet without metatarsalgia: n =30 | For pain group, the maximum plantar force and force time integral of the first metatarsal decrease significantly, the time point when central rays reached their peak force during the push-off is significantly later than that in controls. The regional instant load percentage at this moment presented significantly higher for central rays, while significantly lower for the first metatarsal and the hallux compared to the controls. | [82] |
| **Acute Ankle Sprains and Chronic Ankle Instability** | Plantar pressure test and sEMG | CAI patients: n = 17, Healthy subjects: n = 17 | The CAI group demonstrated a more lateral COP throughout the stance phase and significantly increased peak pressure and pressure–time integral under the lateral forefoot. The CAI group had higher gluteus medius sEMG amplitudes during the final 50% of stance and first 25% of swing. | [86] |
|  | vGRF data were collected using an instrumented treadmill | CAI patients: n =11, Healthy individuals: n = 13 | The CAI group had higher impact peak forces, active peak forces, an increased loading rate and a shorter time to reach the active peak force compared with the control group. | [87] |
|  | Gait analysis,  Musculoskeletal model.  Finite element model | Acute LAS participants n =68, Noninjured participants: n = 19 | During period 1, the LAS group displayed increased knee flexion with increased net extensor pattern at the knee joint, increased ankle inversion with a greater inversion moment, and reduced ankle plantar flexion. During period 2, the LAS group displayed decreased hip extension with a decrease in the flexor moment at the hip, and decreased ankle plantar flexion with a decrease in the net plantar flexion moment. | [88] |
|  | Codamotion marker set model ^[122]^ | Acute LAS: n = 68, Control group: n = 19 | Controls demonstrated greater angles of SAK/TT than individuals with CAI and greater angles of FAK/TT than copers during the second half of stance. | [89] |
|  | Plantar pressure test and sEMG | CAI patients: n = 16 | Gait training improved self-reported function and caused a medial shift in the COP from 10% of stance through toe-off. The medial shift in COP was driven by concurrent increases in peroneus longus muscle activity from 21-60% and 81-90% of stance. There was a corresponding reduction in gluteus medius muscle activity during 71-100% of stance. | [90] |
|  | Plantar pressure test and sEMG | CAI patients were treated with anatomic reconstruction surgery: n = 19 | Dynamic pedography showed a large degree of symmetry of plantar pressure distribution after surgery. There were no significant differences in peroneal reaction time in the repaired and intact ankles. | [92] |
| **Ankle Fracture** | Plantar pressure test and the spatiotemporal gait parameters | Patients with bimalleolar ankle fractures undergoing ORIF: n = 22, Healthy subjects: n = 11 | The main results found in plantar pressure were a lower mean/peak plantar pressure, as well as a lower contact time at 6 and 12 months with respect to the healthy leg and control group and only the control group, respectively. In the ankle fracture groups there are a moderate negative correlation between plantar pressures (average and peak) with bimalleolar and calf circumference. | [99] |
|  | PIG、plantar pressure test and sEMG | Patients with trimalleolar ankle fractures, Healthy subjects: n = 12 | Patients presented compromised gait patterns: shorter step length, larger step width, slower walking speed and shorter single support, and asymmetrical gait. During walking, patients showed abnormal dynamic plantar pressure features (mainly in the hindfoot and forefoot regions), and the IEMG of TA and PL were larger than healthy controls. | [100] |
|  | OFM | Patients operated for ankle fractures: n = 33(33 feet), Healthy control group: n = 11(20 feet) | Significantly less ROM between the hindfoot and tibia in the sagittal plane (flexion/extension) during loading and push-off phases was found in patients after ankle fractures. Lowest ROM and poorest PROM results were found for patients with trimalleolar ankle fractures. | [101] |
|  | HFMM | Patients with isolated ankle fractures: n = 14, Healthy participants: n = 20 | Significant differences for the Foot Tibia Dorsal Flexion, the tibio-talar dorsal flexion and the ground reaction force for patients after 9 weeks as well as patients after 26 weeks compared to healthy participants, respectively. the ROM in the tibio-talar joint and the medial arch was reduced in affected patients compared to healthy participants. | [102] |
|  | RFM | Patients who sustained a trimalleolar fracture and underwent surgery: n = 15, Asymptomatic adults: n = 13 | Mean peak power generation, total positive work and peak internal ankle moment were significantly lower for the Chopart joint when comparing the patients to the control group. These results were observed for both the affected and unaffected side of the patients, showing symmetrical changes in the patient group. | [103] |
| **Ankle Osteoarthritis** | MiFM | DJD group: n = 36, Healthy group: n = 13 | Ankle DJD demonstrates significant changes in foot mechanics characterized by altered segment kinematics and significant reduction in dynamic ROM at the tibia, hindfoot, forefoot, and hallux when compared to controls. The results demonstrate decreased temporal-spatial parameters. | [106] |
|  | Dynamic pedobarography | Posttraumatic end-stage ankle osteoarthritis patients: n = 120 | Maximum force and contact area were decreased in the whole osteoarthritic foot. Peak pressure in the hindfoot and toes area was decreased as well. The results indicated a positive correlation between dorsiflexion and the pedobarographic parameters. | [107] |
|  | A 3D MFM with 15 markers ^[123]^ | Patients undergoing TAR: and AA: n = 17 and 7 | Gait speed was faster in the TAR, the range of hindfoot and forefoot sagittal motion was significantly greater in the TAR. The main component of motion increase was hindfoot dorsiflexion. Maximum ankle power in the TAR was significantly higher than in AA. However, the range of hindfoot and forefoot sagittal motion was decreased in both TAR and AA. | [108] |
|  | mOFM | Patients undergoing TAR: and AA: n = 10 and 10 | During level walking, sagittal ankle ROM was significantly higher, forefoot-tibia motion and hindfoot-tibia motion were significantly greater in the TAA group. During stair ascent, sagittal ankle ROM, forefoot-tibia motion, and hindfoot-tibia motion was greater. | [109] |
|  | MiFM | DJD patients were evaluated before and after TAA: n = 27 | Decreased external rotation of the tibia and increased external rotation of the hindfoot were noted throughout the gait cycle. Ankle replacement as supported by increased temporal-spatial parameters, and significant improvement in tibial sagittal range of motion during terminal stance and hindfoot sagittal range of motion during preswing. | [110] |
